# Simulation and feasibility assessment of a green hydrogen supply chain: a case study in Oman

**DOI:** 10.1007/s11356-024-32563-z

**Published:** 2024-03-02

**Authors:** Mi Tian, Shuya Zhong, Muayad Ahmed Mohsin Al Ghassani, Lars Johanning, Voicu Ion Sucala

**Affiliations:** 1 Department of Engineering, University of Exeter, Exeter, UK; 2 School of Management, University of Bath, Bath, UK; 3 School of Engineering, Computing and Mathematics, University of Plymouth, Plymouth, UK

**Keywords:** Renewable energy, Logistics optimization, Hydrogen economy, Green technology, Energy policy, Decarbonization

## Abstract

The transition to sustainable energy is crucial for mitigating climate change impacts. This study addresses this imperative by simulating a green hydrogen supply chain tailored for residential cooking in Oman. The supply chain encompasses solar energy production, underground storage, pipeline transportation, and residential application, aiming to curtail greenhouse gas emissions and reduce the levelized cost of hydrogen (LCOH). The simulation results suggest leveraging a robust 7 GW solar plant. Oman achieves an impressive annual production of 9.78 TWh of green hydrogen, equivalent to 147,808 tonnes of H_2_, perfectly aligning with the ambitious goals of Oman Vision 2040. The overall LCOH for the green hydrogen supply chain is estimated at a highly competitive 6.826 USD/kg, demonstrating cost competitiveness when benchmarked against analogous studies. A sensitivity analysis highlights Oman’s potential for cost-effective investments in green hydrogen infrastructure, propelling the nation towards a sustainable energy future. This study not only addresses the pressing issue of reducing carbon emissions in the residential sector but also serves as a model for other regions pursuing sustainable energy transitions. The developed simulation models are publicly accessible at https://hychain.co.uk, providing a valuable resource for further research and development in the field of green hydrogen supply chains.

**Supplementary Information:**

The online version contains supplementary material available at 10.1007/s11356-024-32563-z.

## Introduction

The global transition towards sustainable energy has necessitated the exploration of alternative fuels to effectively reduce greenhouse gas emissions and mitigate climate change (Canton 2021). In the residential sector, traditional fossil fuel-based cooking fuels, such as liquefied petroleum gas (LPG), contribute significantly to carbon emissions and air pollution (Singla et al. 2021). As a cleaner and more sustainable alternative, hydrogen has garnered considerable attention due to its high energy content and zero-emission combustion. Hydrogen is one of the most efficient fuels in terms of energy-movement conversion, being approximately 2.5 times more efficient than gasoline (Ball and Wietschel 2009). Moreover, it can be obtained from renewable sources, such as water or biomass, as well as from non-renewable sources, such as coal and hydrocarbon sources. Moreover, hydrogen provides a sustainable way to diversify the energy matrix, ensuring supply safety and enabling easy conversion into electricity whenever needed (Ball and Wietschel 2009).

To use hydrogen as a fuel at a large scale, research and investments are needed to overcome technical, economic, environmental, and structural obstacles (Ferrada et al. 2023). Numerous pilot projects have been developed worldwide to make the adoption of hydrogen feasible. However, the lack of a widespread hydrogen infrastructure poses challenges for its implementation, particularly in the transportation sector.

The hydrogen supply chain encompasses multiple interconnected nodes, including raw material suppliers, production plants, storage points, and applications of hydrogen (Almansoori and Betancourt-Torcat 2016, Degirmenci et al. 2023a, Degirmenci et al. 2023b). The complexity associated with designing an efficient and effective supply chain requires careful consideration of various factors, including production capacity, storage capacity, transportation modes, and demand projections (Almansoori and Betancourt-Torcat 2016). Previous studies have investigated different aspects of the hydrogen supply chain, considering economic, financial, and safety considerations (Nunes et al. 2015; Robles et al. 2020; Santoso et al. 2005). Mathematical models, such as mixed-integer linear programming and stochastic programming, have been utilised to optimise the design and operation of the supply chain (Harichandan and Kar 2023, Bique et al. 2021). The challenge lies in finding an appropriate balance between model detail and computational tractability, which is essential to ensure practical applicability (Riera et al. 2023).

This paper presents a comprehensive case study on the design and application of a green hydrogen supply chain for residential cooking in Oman. Located in the Middle East, Oman is actively pursuing renewable energy goals to reduce its carbon footprint and promote sustainable development (OMAN 2021). The country’s abundant solar resources make it an ideal candidate for harnessing solar power to produce green hydrogen, which can be utilised as a clean cooking fuel in residential settings. Our study evaluates the feasibility and viability of implementing a green hydrogen supply chain for residential cooking in Oman. This involves the design and integration of various phases, including hydrogen production, storage, transportation, and application. By analysing key parameters such as solar power production, storage capacity, pipeline infrastructure, and household energy consumption, this study aims to assess the performance, environmental impact, and economic considerations associated with the green hydrogen supply chain.

Previous research has demonstrated the advantages of hydrogen as a cooking fuel in terms of energy efficiency and emissions reduction (Schmidt Rivera et al. 2018). This highlights the potential of hydrogen as a sustainable and clean alternative for residential cooking. In the context of Oman, the implementation of a green hydrogen supply chain for residential cooking aligns with the country’s renewable energy objectives outlined in Oman Vision 2040. This strategic vision aims to achieve a renewable energy consumption rate of at least 35% by 2040 (OMAN 2021). By leveraging its solar resources and adopting hydrogen as a cooking fuel, Oman can make considerable strides towards achieving its renewable energy goals while simultaneously reducing emissions in the residential sector.

Furthermore, as one of the novel aspects of this research, the developed simulation models are publicly accessible at https://hychain.co.uk, providing a valuable resource for further research and development in the field of green hydrogen supply chains.

In conclusion, this paper addresses the core academic problem of optimising the hydrogen supply chain for residential cooking, a topic that has seen limited exploration in the current literature. Specifically, our research tackles the challenge of implementing a green hydrogen supply chain in a unique geographical and socio-economic context—Oman. By doing so, it not only contributes novel insights into the feasibility and operational dynamics of such a supply chain but also enriches the broader discourse on sustainable energy solutions. This in-depth case study on the design and application of a green hydrogen supply chain for residential cooking in Oman contributes to broader efforts towards achieving sustainable and clean energy solutions. Our work stands out in its approach to integrating various supply chain components with Oman’s specific energy landscape and its public accessibility of simulation models for further research. By evaluating the feasibility, performance, and environmental implications of the green hydrogen supply chain, this study addresses a significant gap in the literature and offers practical, actionable insights for policymakers, researchers, and industry professionals. The adoption of hydrogen as a clean and renewable cooking fuel presents a promising avenue for sustainable cooking solutions, and this study’s findings serve to guide such initiatives.

## Methodology

This work focuses on the green hydrogen supply chain, from production via solar power to underground storage and several storage medium alternatives, pipeline transportation, residential applications (specifically cooking), emissions from the application, and finally, the levelized costs associated with the supply chain. The models are developed in Simulink MATLAB to organise the equations and simulate the supply chain phases. Additional equations are available in the appendices for a more in-depth understanding of the models.

**Fig. 1:**
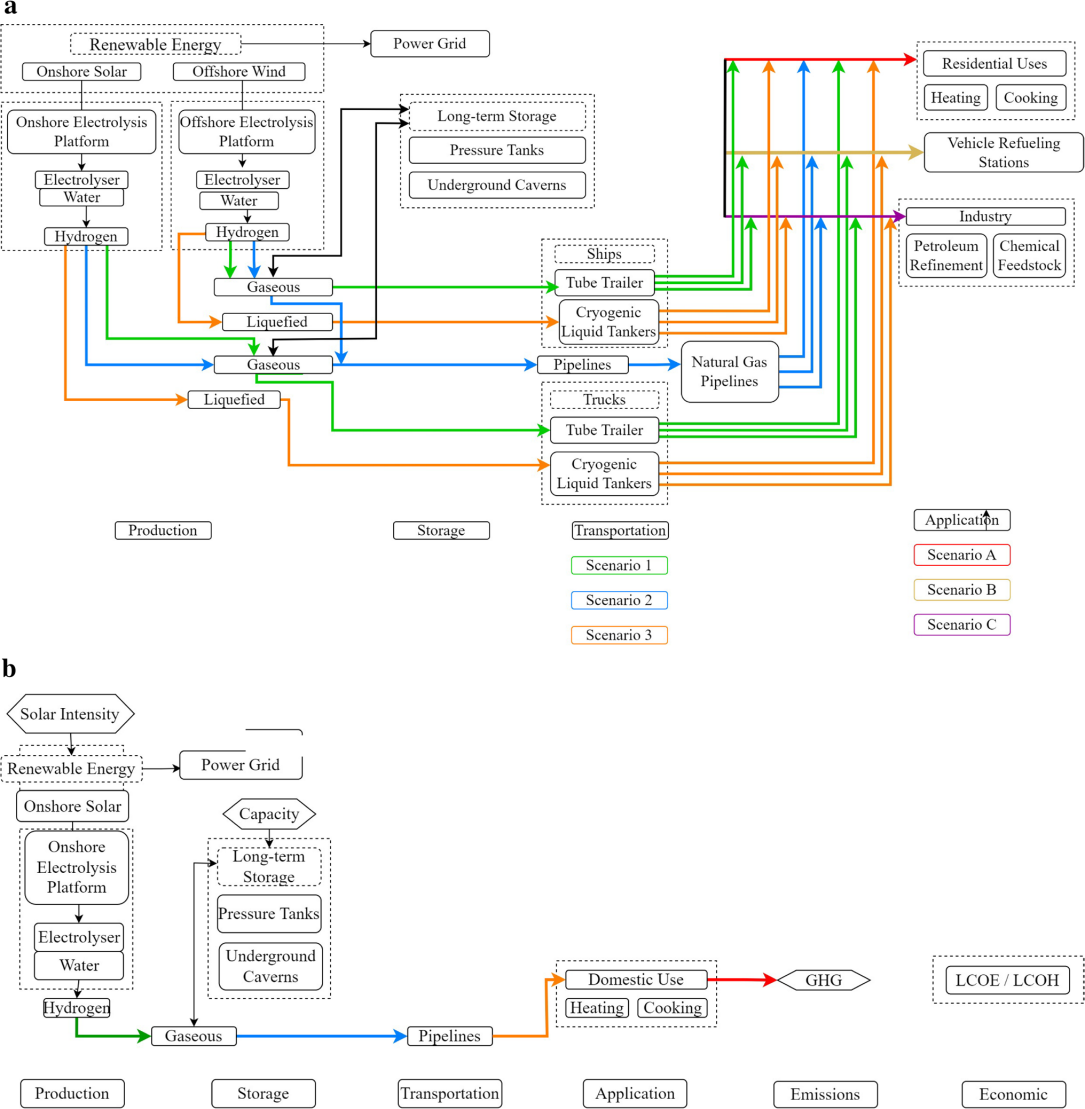
**a** Green hydrogen supply chain flowchart and **b** green hydrogen supply chain flowchart scenario 2A

The green hydrogen supply chain encompasses a range of scenarios in production, storage, transportation, and application, as summarised in Fig. 1. Our analysis focuses on a single, specific scenario. This approach allows for a more in-depth examination and simplifies the flowchart, as depicted in Fig. 1b and Figure S1, providing a clear path for the supply chain.

To select the most suitable scenario for analysis, several factors were considered, such as the regional context, available resources, infrastructure, and potential environmental and economic impacts. Based on these criteria, the chosen scenario consists of the following components:

- Green hydrogen production using solar-powered electrolysis in Oman
- Underground hydrogen storage in the Ghaba salt basin for seasonal use
- Pipeline transportation of hydrogen from Duqm to the Ghaba salt basin
- Residential application of green hydrogen, specifically for cooking purposes

### Algorithm development

Simulink MATLAB has been utilised to construct models that facilitate the organisation of equations and simulation of the diverse phases involved in the supply chain. Figure 3 delineates the model’s focus on the green hydrogen supply chain, commencing with production facilitated by solar power, extending to underground storage, and encompassing various alternative storage mediums. Furthermore, the model encompasses the transportation of green hydrogen via pipelines, its application in residential settings—specifically for cooking—and the consequent emissions resulting from this application. Ultimately, the model incorporates the calculation of the levelized costs correlated with the entire supply chain.

Green hydrogen production necessitates electricity derived from renewable energy sources, such as solar or wind, to power the electrolyser. This dissertation will concentrate on Oman, as delineated in the research aims, given its abundance of solar power, which is harvested more extensively than wind. Consequently, photovoltaic (PV) solar panels will be the chosen electricity production method for green hydrogen in the subsequent model. Furthermore, a proton exchange membrane (PEM) electrolyser will be incorporated into the model owing to its proficiency in managing intermittent power, a characteristic of solar energy. To provide a comprehensive understanding of the model’s structure and its operational dynamics, a detailed flowchart is presented in Figure S2 of the Supplementary Information (SI).

The production model was formulated according to the principles outlined by Ahshan (2021), encompassing Eqs. 1, 2, and 3.

1 $$\mathrm{Solar\;PV\;energy\;output }\left[{\text{kWh}}\right],{E}_{PV}={P}_{r,PV}\cdot {D}_{f}\cdot {P}_{sh}\cdot {N}_{d}$$

2 $$\mathrm{Hydrogen\;production} \left[{Nm}^{3}\right], {H}_{PV,N{m}^{3}}=\frac{{\eta }_{pc}\cdot {E}_{PV}}{{E}_{EL}}$$

3 $$\mathrm{Hydrogen\;production }\left[{\text{kg}}\right], {H}_{PV,kg}=\frac{{H}_{PV,N{m}^{3}}}{\frac{1}{{\rho }_{H2}}}$$

Hydrogen storage represents an effective strategy to mitigate the intermittency characteristic of renewable energy. Among the available solutions, underground storage exhibits considerable potential, largely due to its superior capacity compared to aboveground vessels and its ability to facilitate long-term storage. This attribute proves advantageous for seasonal storage, thereby effectively meeting increased demand. Therefore, the subsequent model will incorporate underground hydrogen storage (UHS).

The storage model, represented by Eq. 4, was formulated in adherence to the guidelines outlined in the referenced studies from Andersson et al. (Andersson and Grönkvist 2019) and Muhammed et al. (Muhammed et al. 2022). The design flow charts are shown in Figures S3 and S4.

4 $${\text{Volume}}=\left[\frac{{H}_{PV}\cdot {P}_{sh,{\text{min}}}}{{\rho }_{H2}} ... \frac{{H}_{PV}\cdot {P}_{sh,{\text{max}}}}{{\rho }_{H2}}\right]$$

Numerous methods exist for the transportation of hydrogen from the production site to storage facilities and points of application, including pipelines, ships, and trucks. However, while trucks are most effective for short-distance transportation and ships are optimal for longer distances while capitalising on sea routes, pipelines represent a viable solution for long-distance terrestrial transportation. Consequently, the ensuing model examines the use of pipelines for hydrogen transportation. Following the principles set out in a 2021 study (Khan et al. 2021), the transportation model is represented by Eqs. 5 and 6. The detailed design of the transport model is shown in Figure S5 in the SI.

$${\text{Pipe}}-\mathrm{system\;levelized\;cost\;of\;hydrogen }\left[{\text{USD}}/{{\text{kgH}}}_{2}\right],$$

5 $${{\text{LCOH}}}_{{\text{Pipe}}-{\text{System}}}={{\text{LCOH}}}_{{\text{Pipe}}}+{{\text{LCOH}}}_{{\text{Comp}}}$$

$$\mathrm{Pipe\;levelized\;cost\;of\;hydrogen }\;[{\text{USD}}/{{\text{kgH}}}_{2}],$$

6 $${{\text{LCOH}}}_{{\text{Pipe}}}=\frac{{{\text{CAPEX}}}_{{\text{Pipe}}}+\mathrm{NonEnergy\;OPE}{{\text{X}}}_{{\text{Pipe}}}}{{\text{Availability}}\cdot {\text{Capacity}}\cdot {d}_{y}}$$

Hydrogen serves a multitude of applications, ranging from transportation fuel to industrial use and extending to residential applications. The use of hydrogen as a vehicular fuel represents a relatively novel application, and currently, few countries possess the requisite infrastructure or capabilities to facilitate it. Furthermore, employing hydrogen in petroleum refining appears to be incongruous in the context of a green hydrogen supply chain, as this application contributes to greenhouse gas emissions. Consequently, this model primarily considers residential applications of hydrogen, specifically as a cooking fuel. This choice is predicated on the fact that in contrast to space heating or cooling—which is contingent on regional requirements—cooking represents a universal application that is a necessity across most countries. Based on the principles outlined in the subsequent source (Rivera et al. 2018), the application model was developed and is encapsulated in Eq. 7. The detailed design of the hydrogen application model is illustrated in Figure S6 in the SI.

7 $$\mathrm{Energy\;demand }\left[{\text{MJ}}\right],\mathrm{E }=\mathrm{ C}\_{\text{p}}\cdot {\text{m}}\cdot\upeta$$

Emissions are intrinsically linked to the application, as they quantify the greenhouse gases (GHGs) emitted from the cooking appliance. Considering the carbon-free nature of hydrogen energy, the model additionally accounts for emissions originating from the storage and transportation stages of the green hydrogen supply chain (Rivera et al. 2018), as detailed in Eqs. 8 and 9. A detailed design flow chart of the emission model is illustrated in Figure S7 in the SI.

8 $$\mathrm{Total\;GHG\;emission }\left[gC{O}_{2}e/kg\right],Em{s}_{Total}=Em{{\text{s}}}_{{{\text{CO}}}_{2}e}\cdot m$$

9 $$\mathrm{GHG\; emission\; }[{\text{g}}/{\text{kg}}],\mathrm{ Em}{{\text{s}}}_{{{\text{CO}}}_{2}e}={{\text{Ems}}}_{{\text{GHG}}}\cdot {\text{GWP}}$$

This model facilitates simulation of the levelized cost of electricity (LCOE) and hydrogen (LCOH) production per kWh and kgH_2_, respectively, incorporating Eq. 10 for LCOE and Eq. 11 for LCOH. The LCOE and LCOH of green hydrogen are evaluated in terms of production and storage. These costs can be combined with the LCOH associated with transportation to ascertain the comprehensive supply chain LCOH. Flow charts detailing the calculations in MATLAB for LCOE and LCOH are depicted in Figures S8 and S9.

10 $$\mathrm{Levelized\;cost\;of\;electricity}$$

$${\text{LCOE}}\;[{\text{USD}}/{\text{kWh}}]=\frac{\left({C}_{PV}\cdot {{\text{CRF}}}_{PV}\right)+\left({C}_{pc}\cdot {{\text{CRF}}}_{pc}\right)+\left({C}_{ins}\cdot {{\text{CRF}}}_{ins}\right)+\left({C}_{mis}\cdot {{\text{CRF}}}_{mis}\right)+{C}_{OM}}{{E}_{PV}}$$

Levelized cost of hydrogen

11 $${\text{LOCH}}\;[{\text{USD}}/{\text{kg}}]=\frac{\left({C}_{EL}\cdot {{\text{CRF}}}_{EL}\right)+{C}_{electricity}+{C}_{HS}+{C}_{OM\_EL}}{{H}_{PV,kg}}$$

### Model validation

After setting up the models in MATLAB, the models used for simulating the hydrogen supply chain were validated. The validation process involved the outcomes generated by our models being compared with the results reported in relevant research papers. To facilitate transparency and reproducibility, Tables S2 to S7 in the SI comprehensively detail all the parameters and their respective values that were employed in this validation process. By recreating the models and comparing the results, the accuracy and reliability of our simulations were ensured. The production model was validated by comparing the annual hydrogen production in Oman, computed by our model, with the results from a previous study. Our model closely aligned with the reported production values (Figure S10), confirming its suitability for application in different scenarios.

The storage model was validated by simulating bottle volume within a storage tank. By replicating the pattern observed in the original study, our model demonstrated its adaptability and potential for simulating hydrogen storage, as shown in Figures S11 and S12. Minor modifications, such as integrating values from the production model and implementing specific operating conditions, can further enhance the model’s accuracy.

The transportation model was validated using data on pipe diameter and distance. The results from our model matched the expected values in Figure S13, indicating its validity for simulating hydrogen transportation. The model’s emphasis on a singular pipe diameter, along with cost variations based on distance, provided reliable insights into the transportation process.

Furthermore, the application model was validated by comparing the energy quantity of different fuels required to meet demand. The results showed that the required quantity of hydrogen was less than that of natural gas, aligning with previous findings, as shown in Figure S14. By leveraging the production model, our application model facilitated effective comparisons, enabling us to determine the number of households that could be satisfied or the capacity of a solar farm to support specific energy demands.

The emission model was also validated by comparing greenhouse gas emissions from hydrogen and natural gas. The results in Figure S15 demonstrated that hydrogen emitted significantly fewer emissions, validating its environmental benefits. However, emissions from the compression and transportation phases remained noteworthy, emphasising the importance of utilising renewable sources for green hydrogen production.

In the final phase of our analysis, a rigorous validation process was conducted on the levelized cost model, specifically focusing on its calculations regarding the LCOH. This validation was further substantiated by the alignment of our simulated LCOH values, as depicted in Figure S16, with those documented in prior studies, thereby reinforcing the model’s reliability across various scenarios. Overall, this comprehensive validation process not only confirms the accuracy of our models but also supports their application in exploring different dimensions of the hydrogen supply chain.

## Result analysis

The overarching objective of this work is to leverage the developed models to simulate the green hydrogen supply chain within a case study based in Oman, thereby aligning with the objectives of Oman Vision 2040. One of the primary goals of this vision is to achieve a renewable energy consumption rate that constitutes at least 35% of total consumption by 2040, up from the current rate of 0% (OMAN 2021). To that end, the subsequent case study parameters and assumptions are tailored to fulfil this target, while the unmentioned factors remain consistent with the validation section.

**Fig. 2:**
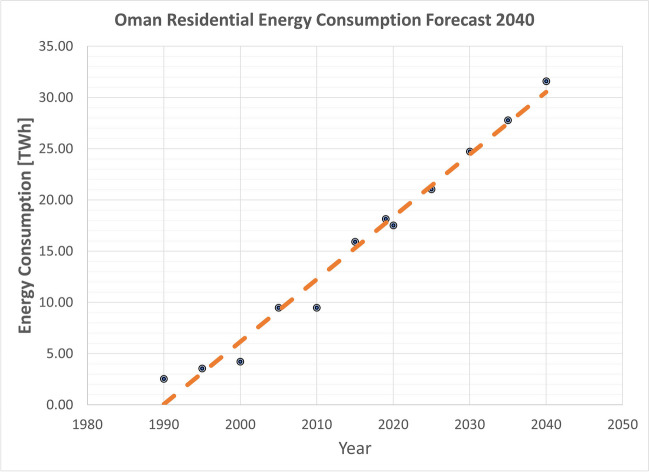
Oman residential energy consumption forecast 2040

As reported in 2019, Oman’s energy consumption via residential uses was 18.16 TWh, while its renewable energy supply amounted to 3.89 GWh (IEA 2019). However, linear forecasting, as depicted in Fig. 2, indicates that residential energy consumption will reach 31.59 TWh by 2040. This forecast has profound implications for Oman’s energy landscape. To meet the ambitious target of achieving a 35% renewable energy consumption rate in the residential sector by 2040, there is a pressing need for an additional annual production of 11.05 TWh of energy from renewable sources. This sizable increase is necessary to align with Oman’s sustainability objectives and address the escalating energy demand in the residential sector.

While the hydrogen production model can be simplified by selecting the location with the highest energy and hydrogen production, it is crucial to consider other complexities, such as accessibility to underground storage and proximity to densely populated residential areas. These factors can help reduce costs associated with hydrogen storage and transportation. In Oman, three major salt basins exist that can host salt caverns for hydrogen storage: Fahud, Ghaba, and South Oman (Thomas et al. 2015). It is also important to note that the cities with the highest populations in Oman are concentrated in the north (World-Population 2022).

However, another challenge with solar plants is their requirement for large amounts of vacant land, which can be difficult to find near densely populated cities. As a result, priority should be given to locations with high solar irradiation and those closer to salt basins. As a result, Duqm has been chosen as the focal point of this work due to its high solar irradiation, ranking second after Marmul. Additionally, Duqm’s unique geographic location on the coast in central Oman enables it to serve the north, south, and even sea routes, while being in proximity to two salt basins, Ghaba and South Oman. Furthermore, Duqm is classified as a special economic zone in Oman and thus presents greater potential for project development and investments.

**Fig. 3:**
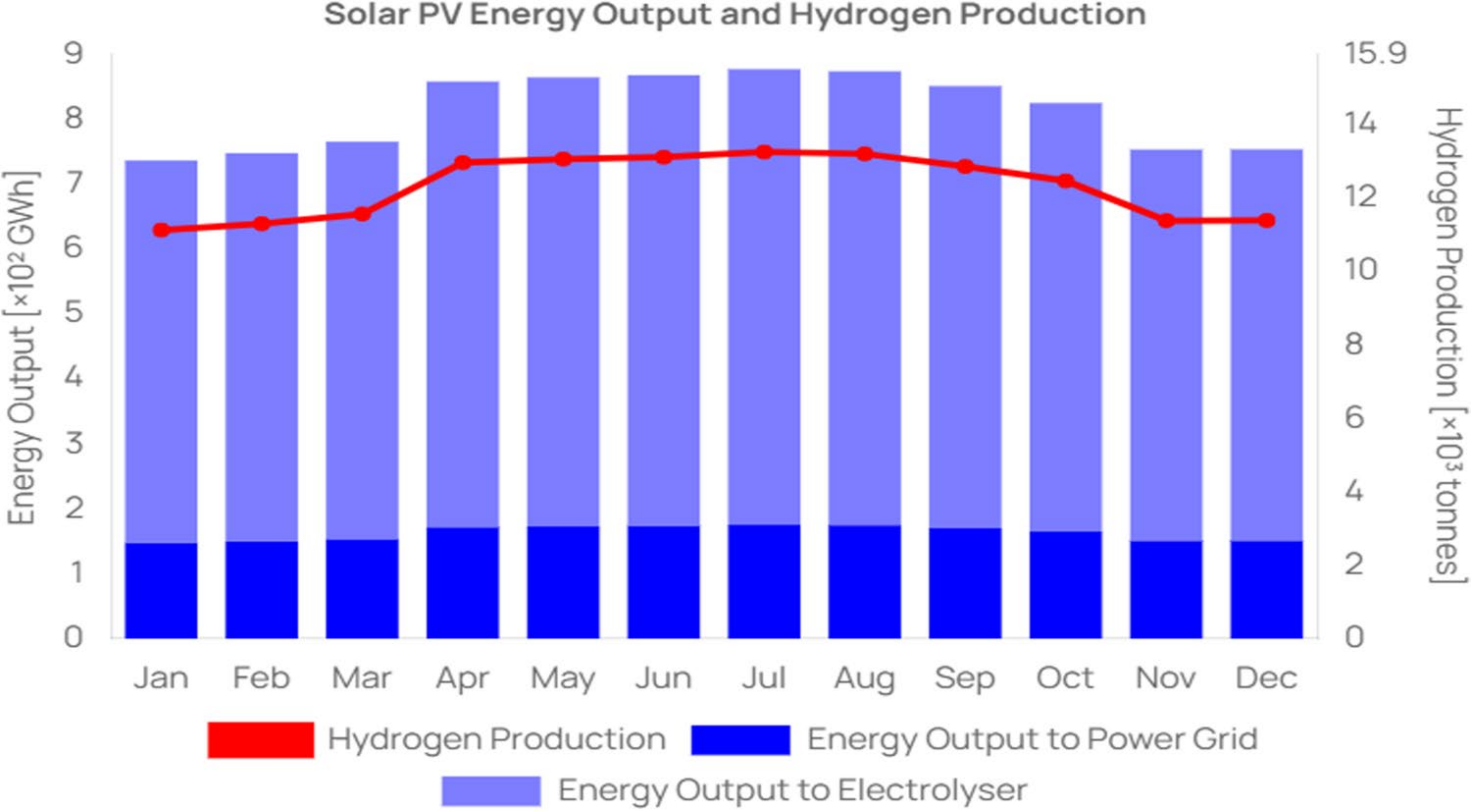
Monthly average hydrogen production in Duqm

The total annual energy output from the proposed 7 GW solar plant is projected to be approximately 9.78 TWh, which equates to approximately 147,808 tonnes of hydrogen, as shown in Table 1. This estimate takes into account variations in production that occur throughout the year, as illustrated in Fig. 3. Examining monthly data, we observe that the lowest production occurs in January, with an output of approximately 0.7369 TWh, equivalent to approximately 11,137 tonnes of hydrogen. On the other end of the spectrum, July boasts the highest production levels, yielding approximately 0.8779 TWh, or approximately 13,268 tonnes of hydrogen. This fluctuation in production throughout the year is a critical consideration for planning and optimising the hydrogen supply chain. Figure 3 provides a visual overview of these monthly average hydrogen production figures in Duqm.

**Table 1:** Solar irradiation variation by month parameters in Duqm

Parameter	Value	Reference
${P}_{r,PV}$	7 GW	Assumed
$N{a}_{s}$	[Jan, Feb, Mar, Apr, May, Jun, Jul, Aug, Sep, Oct, Nov, Dec]	(Al-Hatmi et al. 2016, PACA 2020)
${P}_{sh}$	[4.59, 4.67, 4.80, 5.42, 5.50, 5.53, 5.58, 5.54, 5.39, 5.21, 4.73, 4.71] h/day	
${T}_{a}$	[20.9, 22.1, 24.9, 29.5, 34.2, 35.2, 34.1, 31.9, 31.2, 29.4, 25.6, 22.6] ℃	
${N}_{d}$	30.42 days	Assumed

The simulation shows significant monthly variation in hydrogen production, directly correlated with solar irradiance levels. In Oman, solar irradiance peaks during the summer months, leading to increased hydrogen production. This trend aligns with the findings of Al-Hatmi et al. (2016), who noted similar patterns in solar energy potential in the Middle East. The physical principle here is the direct relationship between solar irradiance and the efficiency of photovoltaic (PV) panels used in hydrogen production.

To simulate UHS in Oman, a suitable storage capacity must be determined. As previously stated, Oman possesses three salt basins situated in the northern, central, and southern regions. Duqm, given its proximity to these basins, can potentially benefit from local storage facilities. These salt basins present a feasible opportunity for the construction of salt caverns, which could serve as large capacity storage facilities, particularly for seasonal storage.

**Fig. 4:**
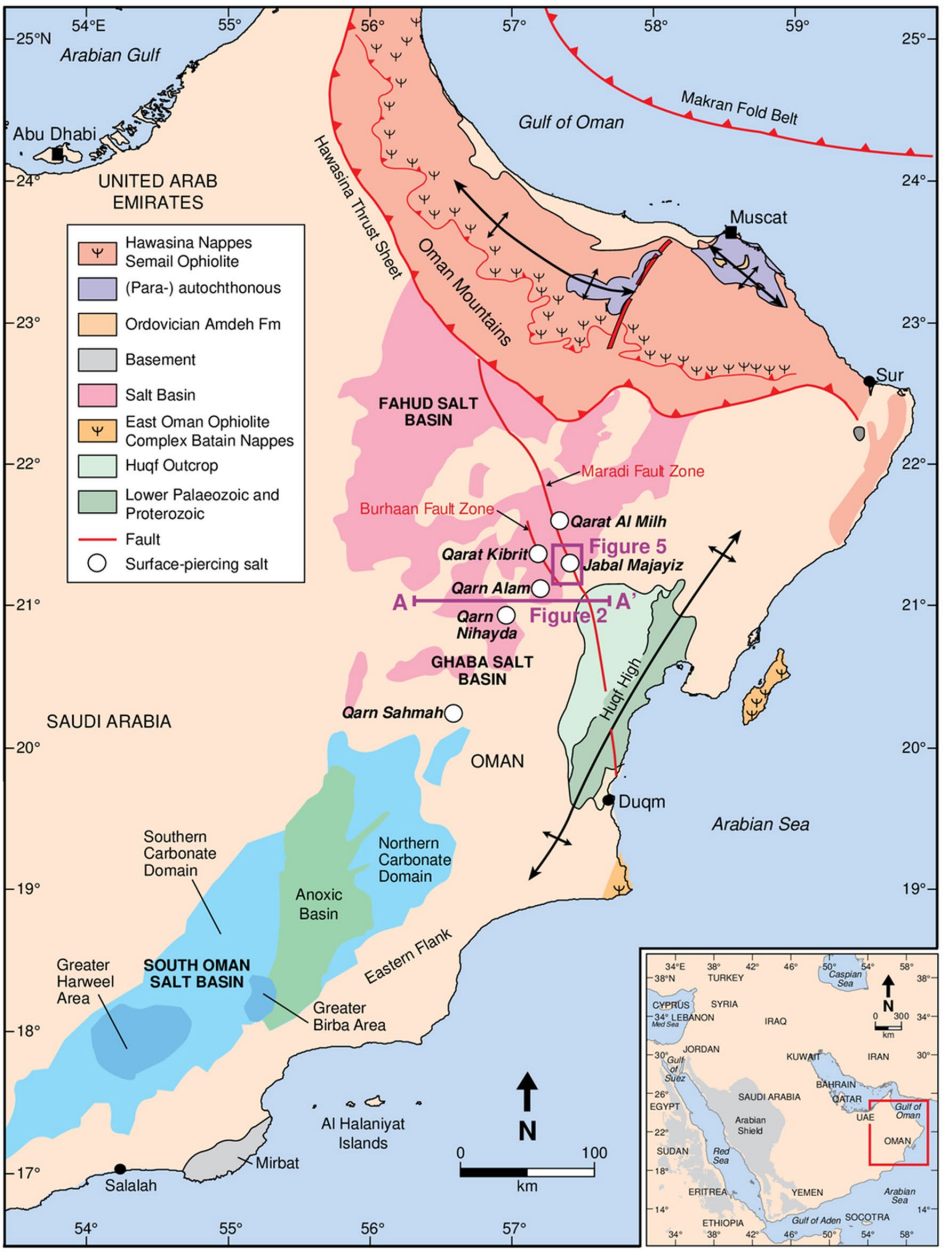
Salt basins in Oman (Reuning et al. 2009)

Figure 4 illustrates the distribution of salt basins across Oman, with a focus on the Ghaba salt basin marked with specific ‘surface-piercing salt’ locations. These salt domes have distinct specifications, as detailed in Table 2. The maximum volume of these salt domes is estimated based on the geometric resemblance to an ellipsoid.

**Table 2:** UHS parameters

Parameter	Value	Reference
${P}_{sh,{\text{min}}}$	80%	(Ahshan 2021)
${P}_{sh,{\text{max}}}$	113%	
${Vol}_{Str}$	1,000,000 ${{\text{m}}}^{3}$	(Crotogino 2022; Peters et al. 2003)
${P}_{Str}$	45 bar	(Muhammed et al. 2022)
${T}_{Str}$	50 ℃	
${N}_{d}$	1 day	Assumed
${t}_{st}$	2 days	Assumed
${\rho }_{Med}$	[123, 99, 84, 84, 70, 14.7]${{{\text{kgH}}}_{2}/{\text{m}}}^{3}$	(Andersson and Grönkvist 2019)
${{\text{LHV}}}_{{H}_{2}}$	120 MJ/kg	(Rivera et al. 2018)

The selected storage volume is set at 1 × 10^6^ m^3^, which corresponds to the upper limit capacity of typical salt caverns (Crotogino 2022). This volume is less than that of the salt domes in the Ghaba Salt Basin, indicating that the construction of such a cavern is feasible. A 1-day storage period is assumed for the simulation to reflect daily variations throughout the year. Meanwhile, the release time is presumed to be 2 days to represent the fill-up cycle typical for seasonal storage.

**Fig. 5:**
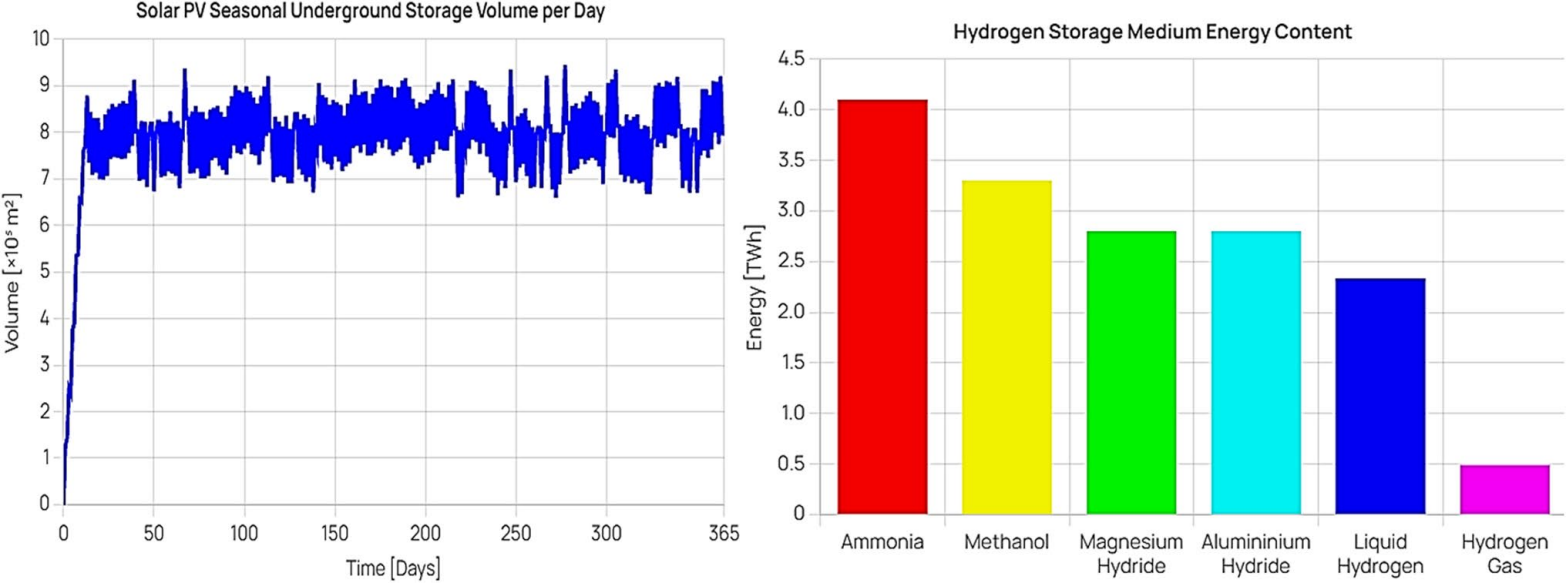
(left) Storage case study UHS volume variation and (right) alternative hydrogen storage medium comparison

The simulation result, depicted in the left side of Fig. 5, demonstrates that approximately 13 days are needed to fill the underground storage cavern, given the production rate from the previous stage. This offers a significant advantage, as the stored hydrogen can be conserved for prolonged periods until demand rises. This effectively resolves the intermittency issue associated with renewable energy, facilitating more efficient and cost-effective energy storage.

The variation in volume observed after the cavern is filled is due to hydrogen being alternately released and pumped back in at different volumes owing to fluctuations in production. This aspect augments the simulation’s realism, as hydrogen production, especially from solar power, is subject to variation based on multiple factors.

Different storage media have been simulated, with the results presented on the right side of Fig. 5. These simulations maintain the constant operating conditions established during the model validation phase. The comparison takes into account the volumetric density of each medium to calculate the amount of energy that can be stored for a specific storage volume. The graph follows the trend observed in the validation results, demonstrating that ammonia—with its high energy density—can store the most energy, while gaseous hydrogen stores the least. Ammonia may emerge as a cost-effective option for storage, given the lower costs associated with its compression and storage. However, its use may be limited in applications requiring very high purity hydrogen, such as fuel cells. In such cases, the hydrogen extracted from ammonia must be highly purified to avoid catalyst poisoning and potential damage to the fuel cell. Therefore, a comprehensive analysis considering all these factors is crucial to choosing the most suitable hydrogen storage medium for a particular application or scenario.

Our model suggests that underground hydrogen storage in the Ghaba salt basin is feasible and efficient for seasonal demand management. This finding resonates with Andersson and Grönkvist (2019), who demonstrated the high efficiency and capacity of underground hydrogen storage. The underlying physical principle is the high volumetric energy density of hydrogen, which makes underground storage a viable option for large-scale energy storage.

**Fig. 6:**
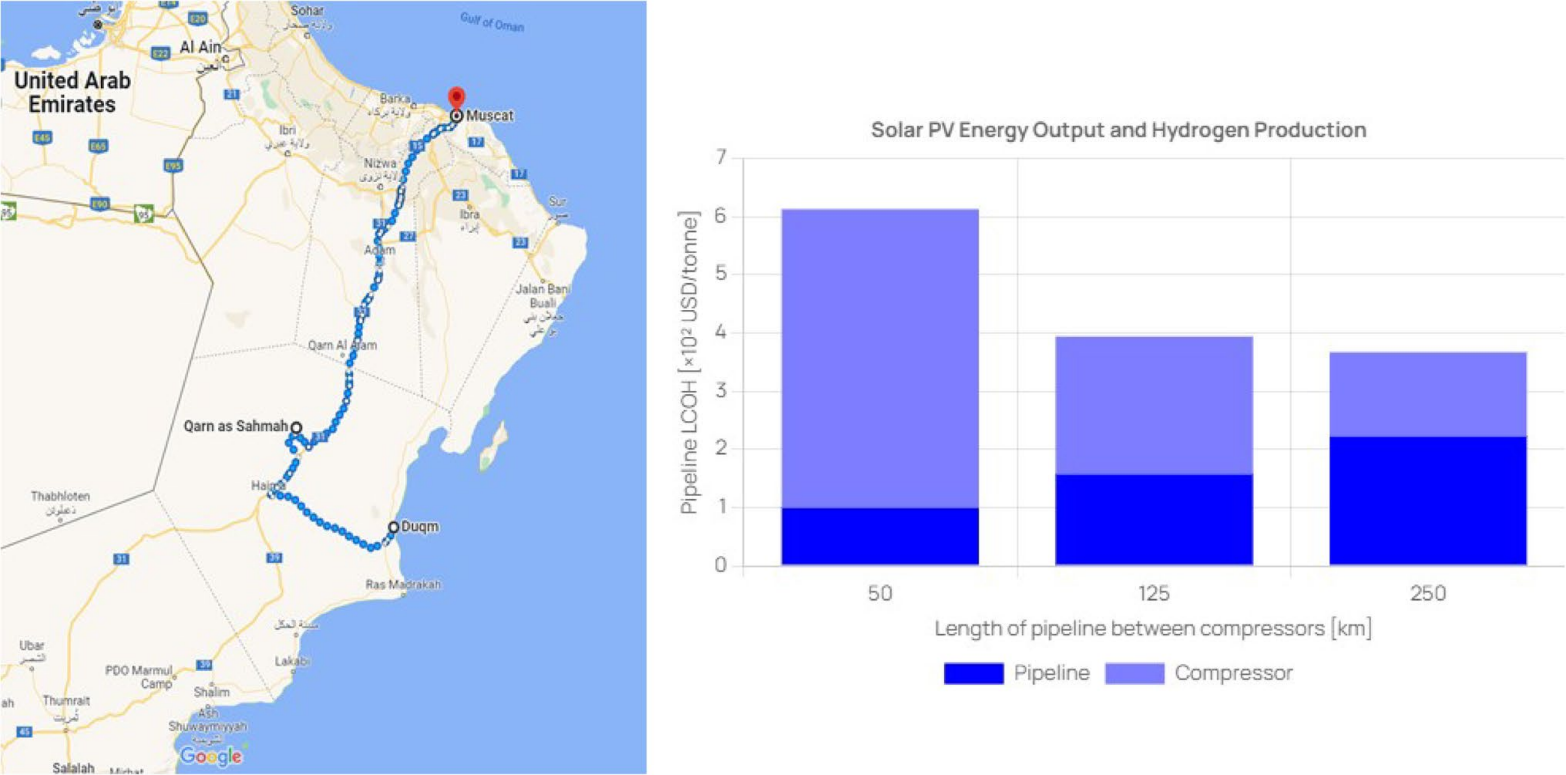
(left) The proposed hydrogen pipeline system in Google Maps, and (right) LCOH for the pipeline system vs. the length of pipeline between compressors

Pivoting from storage, it is essential to contemplate the strategic aspect of hydrogen transportation within the context of Oman. Oman’s industrial sector already boasts an established network of pipelines, primarily designed to facilitate the transport of oil and gas from extraction sites to refineries and ports. These pipelines are predominantly located inland, aligning with the distribution of oil and gas fields, and stretch out towards coastal regions where the main refineries and ports are situated. In scenarios pertaining to the industrial utilisation of hydrogen, it could be feasible to repurpose these existing pipelines to convey hydrogen to refineries. As this work focuses on residential applications, it necessitates the consideration of a newly installed and operated pipeline system. This proposed pipeline system is projected to span from the hypothetical hydrogen production facility at Duqm to the underground storage structure situated within the Ghaba salt basin and further extend to densely populated areas in the northern regions, as shown in the light side of Fig. 6.

The total distance covered by the pipeline is estimated to be approximately 750 km, as indicated by the route in Google Maps in the left side of Fig. 6. Thus, options are being explored involving the placement of compressors every 50, 125, and 250 km, given their divisibility by the total pipeline distance. The chosen diameter for the pipe is 36 inches, which aligns with the maximum diameter found in Oman’s gas pipelines (CARMEN 2021). This corresponds to an internal diameter (*D*_in_) of 895.3 mm. The cost of electricity (*C*_Elc_) used for compression is assumed to be 0.059 USD/kWh, as referenced from (Ahshan 2021). The LCOH for the pipeline system, presented in the right side of Fig. 6, elucidates the cost implications of different compressor station arrangements designed to sustain the requisite flow rate and pressure. With compressor stations situated every 50 km, a total of 14 stations would be necessary, resulting in costs more than double those associated with a 250 km spacing. Moreover, when comparing compressor placements every 125 km to every 250 km, a cost difference of approximately 24% is observed. Therefore, a configuration featuring two compressor stations spaced 250 km apart appears to strike an optimal balance between cost-effectiveness and ease of maintenance.

Transitioning from the pipeline system’s design and optimization, the focus shifts to the application of transported hydrogen, with a specific emphasis on its residential usage. To ensure consistency, the parameters for hydrogen and natural gas established during model validation are retained. Parameters for liquefied petroleum gas (LPG), the primary fuel for cooking in Oman, are concurrently introduced. By juxtaposing these fuels, a comprehensive understanding of their relative performance in this context is provided. According to the data provided by the National Centre for Statistics and Information (NCSI 2018), the number of households in Muscat, Oman, stood at 89,312 in 2018. A linear projection estimates that this figure will reach 156,307 by 2040. In accordance with the values provided by Rivera et al. (Rivera et al. 2018), the average daily energy consumption for cooking is 9 MJ. The energy efficiency of LPG is 60%, and its net calorific value (NCV) is 46.1 MJ/kg. These parameters serve as the basis for our simulation of energy usage and a comparison of different fuels within the context of residential cooking applications.

**Fig. 7:**
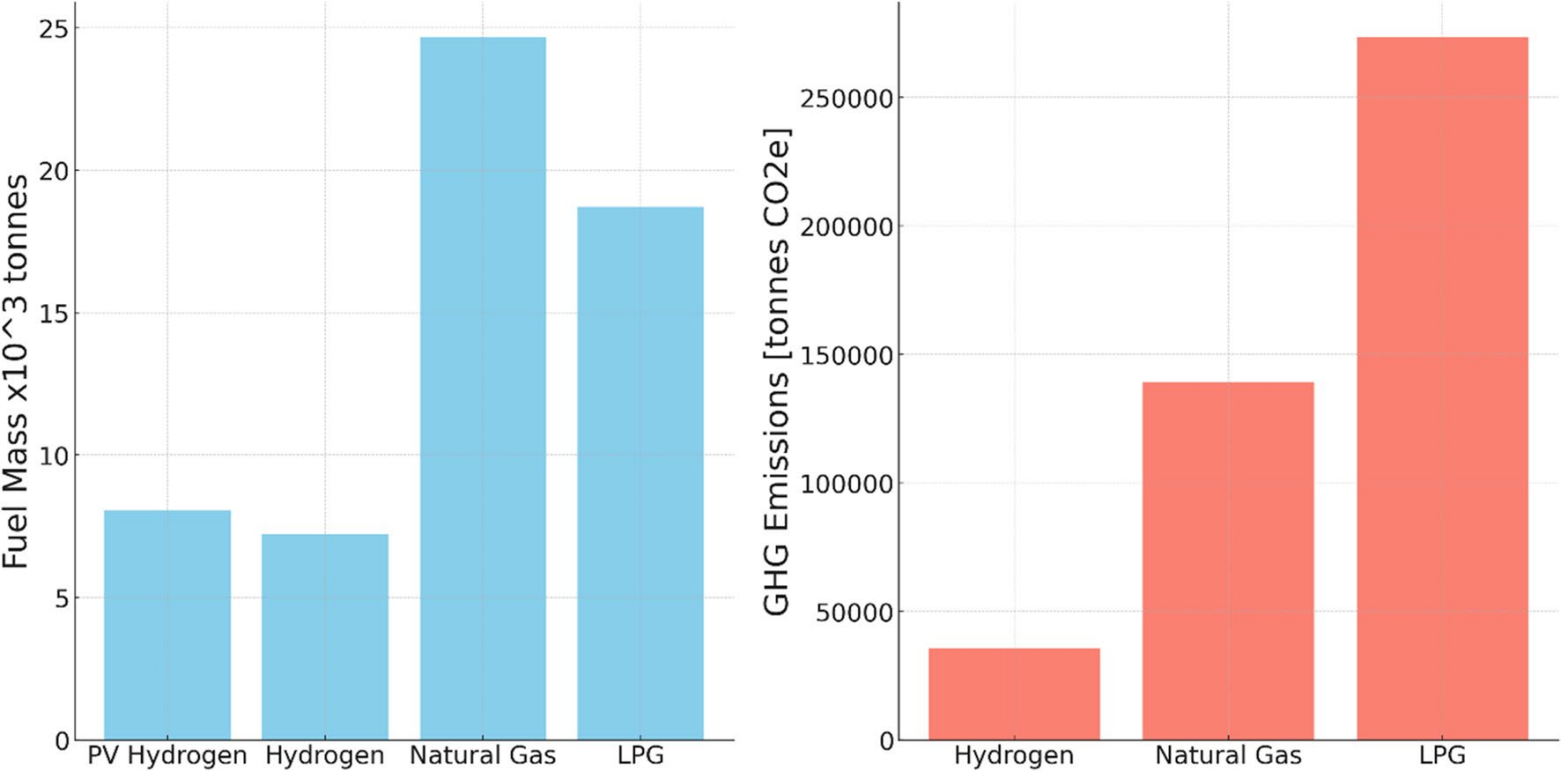
(left) The fuel required to meet the energy demand for cooking across 156,307 households in Muscat, Oman, based on a 300 MW plant, and (Right) emissions from the different fuels

The left figure in Fig. 7 presents the quantity of fuel required to meet the energy demand for cooking across 156,307 households in Muscat, Oman. Given the significant surplus produced by a 7 GW solar plant—exceeding energy requirements by more than a factor of 20—the reduced capacity of a 300 MW plant is used for the simulation. This generates a quantity of hydrogen more commensurate with the anticipated demand.

In terms of raw material consumption, an estimated 18,500 tonnes of LPG is juxtaposed with 7000 tonnes of hydrogen. This comparison suggests that hydrogen could offer a more efficient alternative to LPG for cooking, as it requires approximately 60% less fuel to generate the same amount of energy. Nevertheless, other factors demand consideration, including fuel availability, generated emissions, and cost implications. Given the current calculations, a 300 MW solar plant appears capable of meeting the demand. The subsequent sections will provide a detailed exploration of the emissions and costs of this supply chain.

Consistency with the validation section is maintained by retaining the parameters for the emissions model. However, for comparative analysis, GHG emissions of LPG are introduced alongside green hydrogen and natural gas. The following emissions data (Department of Energy, DOE (2022)) are included: ${{\text{Ems}}}_{{{\text{CO}}}_{2}/{\text{LPG}}}$ 2721 g/kg, ${{\text{Ems}}}_{{{\text{CH}}}_{4}/{\text{LPG}}}$ 1: 34.1 g/kg, and ${{\text{Ems}}}_{{{\text{N}}}_{2}{\text{O}}/{\text{LPG}}}$ 28.74 g/kg.

The right bar chart in Fig. 7 elucidates the emissions from LPG, hydrogen, and natural gas. LPG generates the highest emissions, approximately 270,000 tonnes of CO_2e_ annually, to meet the cooking demand outlined in this case study. In contrast, hydrogen produces the least emissions, approximately 35,000 tonnes of CO_2e_. This supports the environmental sustainability claims made by Rivera et al. (2018). Notably, as green hydrogen is derived from renewable sources, its production does not directly result in GHG emissions, and its combustion is carbon-free. However, PV cells, instrumental in hydrogen production, incur indirect emissions across their lifecycle (Yıldız et al. 2020). Consequently, the supply chain emissions attributable to hydrogen are associated primarily with the production, storage, and transportation stages. Given these findings, it is evident that hydrogen is a superior cooking fuel alternative to natural gas and LPG in terms of emission generation, thereby underlining its environmental advantages.

The model-derived levelized costs are 58.4 USD/MWh (or 0.0584 USD/kWh) for the LCOE and 6.37 USD/kg for the LCOH. The calculation of LCOH includes the costs of production and a storage rate of 0.5 USD/kg. The cost of the pipeline in the 250 km pipeline case is set to 456 USD/tonne (or 0.456 USD/kg). Consequently, the total LCOH for green hydrogen—produced in Duqm, stored in the Ghaba Salt basin, and transported to Muscat for residential cooking applications—is 6.826 USD/kg. This is in line with the findings of Yan et al. (2021) and Franco et al. (2021), who reported similar costs in different geographical contexts. The economic viability of green hydrogen, as highlighted in these studies, stems from the decreasing costs of renewable energy technologies and the scale-up of hydrogen production facilities.

The ‘One-Factor-At-A-Time’ method is employed to assess the sensitivity of the developed models, enabling the identification of parameters exerting the greatest influence on the results. In this analysis, parameters are adjusted by ± 50% of their default value from the case study to illustrate the subsequent changes in the output.

Within the production model, alterations were made to parameters such as the solar plant capacity, peak sun hours, and ambient temperature to evaluate their impact on hydrogen production. The modifications applied to the top two parameters—solar plant capacity and peak sun hours—revealed a linear relationship with the model output and exerted the most substantial effect. Conversely, variations in ambient temperature displayed a much more subdued impact on hydrogen production. Notably, the ambient temperature parameter exhibited an inverse relationship: an increase in temperature reduced the output. This result is logical as higher ambient temperatures are known to negatively impact the efficiency of solar cells.

**Fig. 8:**
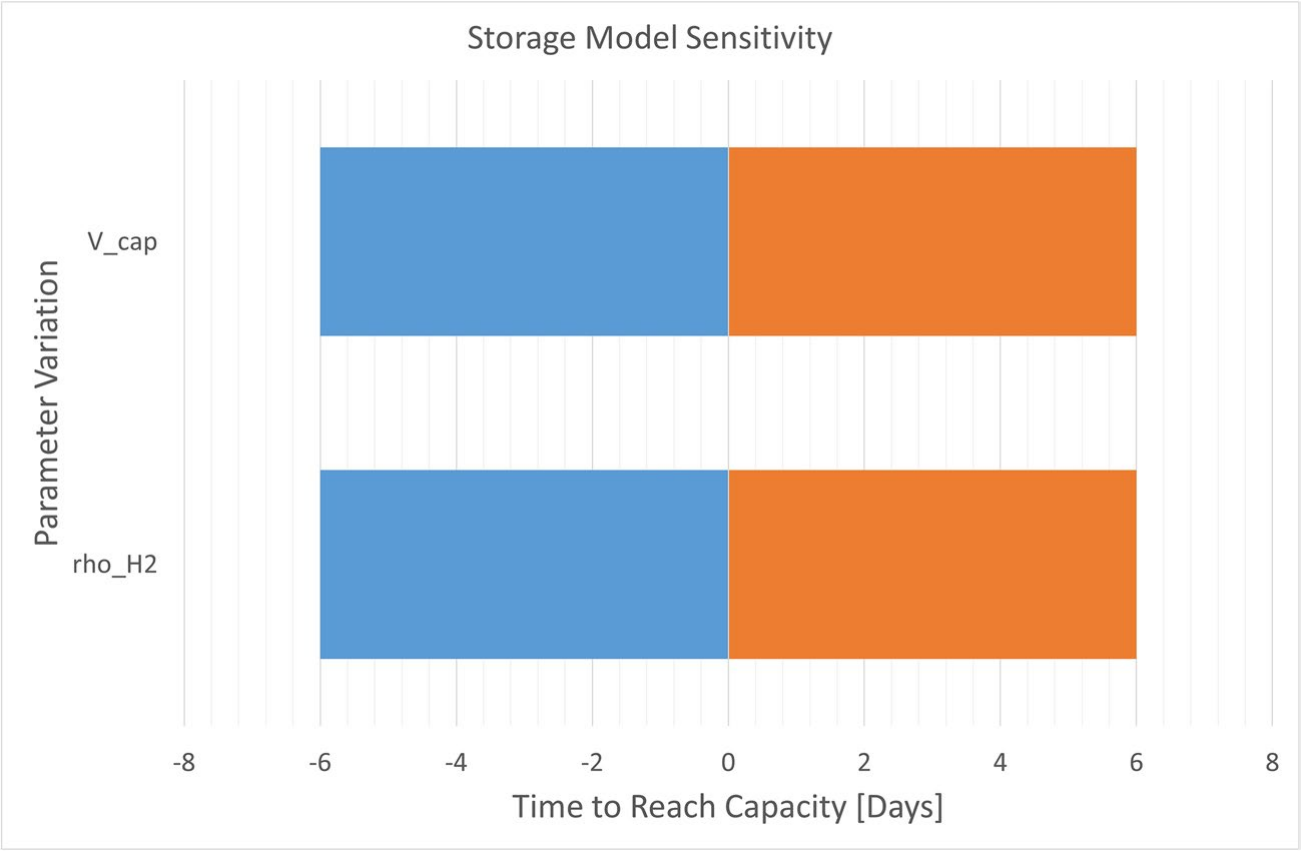
Tornado chart for the storage model

Similar trends were observed in the storage model, with analogous variations in the time required to reach capacity when altering the volume capacity and hydrogen density parameters, given the operating conditions. However, the model accommodates an arbitrary fluctuation in production within the range of 80–113%, accounting for variable weather conditions throughout the year. Consequently, the time required to reach capacity also exhibits variation in each simulation, ranging from 2 to 6 days. This maximum variation is captured in the tornado chart in Fig. 8.

Given the various influencing factors, the transportation model displays nonlinear relationships. This means that an increase in a parameter does not necessarily lead to a proportional decrease in output, and vice versa. The total pipe length exhibits the highest sensitivity, closely followed by the pipe diameter, which is logical given that these changes impact the entirety of the pipeline system and represent its primary components.

The distance between compressors, represented by *L*_pipe_, displays intriguing sensitivity dynamics. While increasing this distance modestly lowers the cost, reducing it significantly increases the cost. This can be attributed to the requirement for more compressors when they are positioned closer together, leading to increased costs related to installation, operation, and energy consumption. Changes in the material base cost demonstrate the least sensitivity, as this affects only the one-time cost of pipe installation.

The application model reveals that the most sensitive factors are the efficiency and the fuel’s net calorific value. Increasing these parameters reduces the fuel required but decreasing them necessitates a larger proportion of fuel. This is because both are dividend variables, and a 50% variation does not correspond to an identical proportion of overall output change. The number of households and their consumption levels display a proportional relationship, exerting an equal effect on the fuel required.

The emission model sensitivity analysis, in descending order, identifies nitrous oxide, methane, and carbon dioxide emissions. This ranking is underpinned by the respective global warming potential (GWP) of these gases, with nitrous oxide displaying approximately 300 times the GWP of carbon dioxide. Consequently, an identical mass of these gases would absorb substantially more energy, leading to a higher rate of global warming.

The levelized cost model, which is intrinsically linked to the production model, identifies the quantity of hydrogen produced as the most sensitive parameter. This parameter exhibits a nonlinear relationship, wherein an increase in production leads to a decrease in LCOH, owing to the increased abundance, thereby making it more cost-effective. The cost of electricity, which is predicated on hydrogen production, and the installation cost are reaffirmed as the dominant factors influencing LCOH (Ahshan 2021). Costs associated with the installation of the PV plant, operation of the electrolyser, and hydrogen storage influence the model’s sensitivity, in that order. On the other hand, the cost of water for electrolyser operation exhibited no significant impact from a 50% variation. This is primarily because its influence is overshadowed by other interrelated factors that collectively determine the LCOH.

## Discussion

The production model reveals that the period of highest hydrogen production coincides with the summer months, which is due to the increase in peak sun hours. Conversely, production is lower during the winter months. Residential energy use, which involves space heating, water heating, and cooking, presents a varying consumption pattern. While water heating and cooking remain fairly consistent throughout the year, space heating sees a significant uptick during the colder winter months (Aras 2008). This pattern can be leveraged to store hydrogen in the summer months, when production exceeds consumption, for use in the winter months, when production is lower. However, in the case of Oman, where space cooling is more prevalent than heating, the excess electricity generated during the summer could be channelled into the power grid to meet the higher cooling demands.

The storage model demonstrates that the time required to reach capacity is contingent on the UHS capacity and the operating conditions. In the scenario where gaseous hydrogen is stored under 45 bar and 50 ℃ in a 1 million m^3^ storage facility, it takes approximately 13 days to reach capacity, given hydrogen production from a 7 GW PV plant located in Duqm. This setup is well suited for seasonal storage, providing a buffer for periods of high energy demand. For example, Oman experiences higher energy consumption during the summer due to the increased demand for space conditioning. The excess electricity produced by solar panels can either be supplied to the grid or utilised to produce hydrogen. The choice between these two options depends primarily on the comparative costs of the transportation and storage of hydrogen. Furthermore, the type of storage medium can be selected based on its volumetric density, e.g., ammonia, providing a space-saving solution and enabling cost-effective storage under reduced operating conditions.

Transportation-wise, hydrogen can be pipelined from Duqm to the Ghaba salt basin for storage and then to Muscat for residential use. This process would involve a total pipeline length of 750 km, with two compressor stations installed every 250 km to maintain the flow rate and operating conditions. The estimated LCOH for this setup is 0.456 USD/kg. However, there are existing gas pipelines between the Ghaba salt basin and Muscat that could be optimised for hydrogen (Droste 1997). Further studies are needed to assess the technological and economic feasibility of this option.

In terms of application, hydrogen can deliver the same amount of energy as LPG with 60% less fuel. However, given the lower volumetric density of hydrogen, a larger storage tank would be necessary. Additionally, shifting to hydrogen as a cooking fuel would entail changes in safety management procedures and the need for domestic appliance upgrades. Therefore, the decision to use hydrogen as a cooking fuel would hinge on the individual user’s priorities and their willingness to accommodate these changes.

The government and local authorities have a crucial role in driving the transition to hydrogen as a cooking fuel through investments and policy initiatives. By providing financial support and incentives, such as grants or subsidies, they can encourage the adoption of hydrogen technologies and infrastructure development. Additionally, implementing supportive policies, regulations, and standards can create a favourable environment for the safe and efficient use of hydrogen in residential cooking applications. These measures can include establishing guidelines for appliance upgrades, promoting research and development in hydrogen technologies, and setting targets for renewable energy integration. By actively engaging in the transition to hydrogen, the government and local authorities can play a significant role in facilitating the widespread adoption of hydrogen as a clean and sustainable cooking fuel.

From an environmental perspective, hydrogen is a cleaner alternative to LPG, producing 87% less GHG emissions throughout its supply chain. This is because hydrogen combustion is carbon-free, but the energy required to store and transport it under certain operating conditions does contribute to its carbon footprint. While hydrogen has the lowest emissions among cooking fuel alternatives, it can have higher impacts in terms of other environmental aspects, such as metal depletion, water eutrophication, and ecotoxicity.

The levelized costs of electricity and hydrogen from the PV plant in Duqm are estimated to be 0.0584 USD/kWh and 6.37 USD/kg, respectively. Including the pipeline transportation cost of 0.456 USD/kg, the overall LCOH for the supply chain is 6.826 USD/kg. This cost aligns with the results of other studies conducted worldwide. As illustrated in Table 3, the LCOH values for green hydrogen vary between 2.95 and 7.40 USD/kg according to location, assumptions, and pathways considered. This consistency indicates that the calculated value is reasonable and further reinforces its validity through alignment with scientific studies that have estimated similar costs. The convergence of findings across multiple investigations adds credibility to the accuracy and reliability of the LCOH estimation, bolstering confidence in its applicability and usefulness for evaluating the economic viability of the hydrogen supply chain.

**Table 3:** Levelized cost comparison

Location	Energy source	LCOH [USD/kg]	Reference
Duqm, Oman	Solar	6.826	This work
China	Wind	4.77–4.79	(Yan et al. 2021)
Europe	Wind	5.62–7.40	(Franco et al. 2021)
Europe	Solar	2.95–4.98	(Vartiainen et al. 2021)
Oman	Solar	6.31–7.32	(Ahshan 2021)
Saudi Arabia	Solar	4.90–5.90	(Khan et al. 2021)

## Web application

A free, user-friendly, and interactive web application with graphical user interface (GUI) is derived from the proposed green hydrogen supply chain simulation models. An ‘Electricity to Hydrogen Tool’ is built in the HyChain platform to demonstrate a green hydrogen supply chain to illustrate the economic and environmental performances (accessible via https://hychain.co.uk). Any end-users can carry out hands-on practise in the web application.

**Fig. 9:**
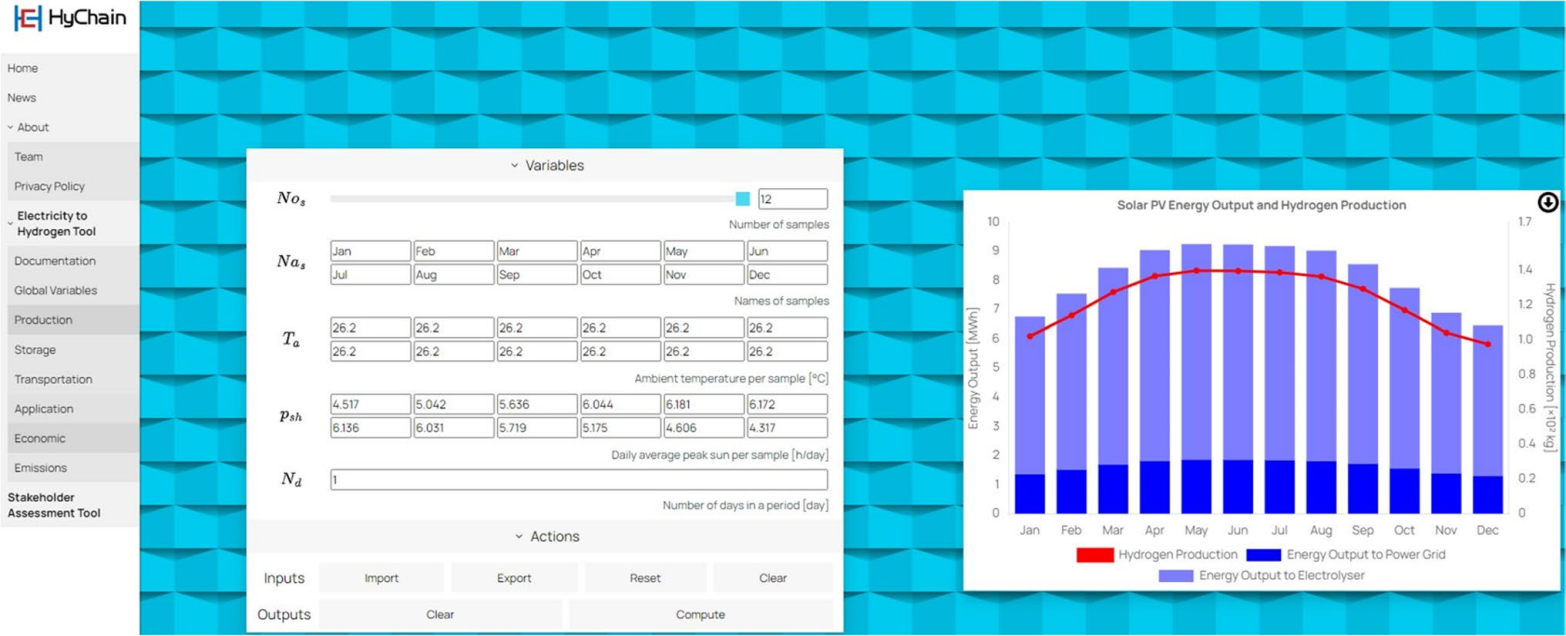
Web application ‘Electricity to Hydrogen Tool’ built in HyChain platform (Production subpage)

**Fig. 10:**
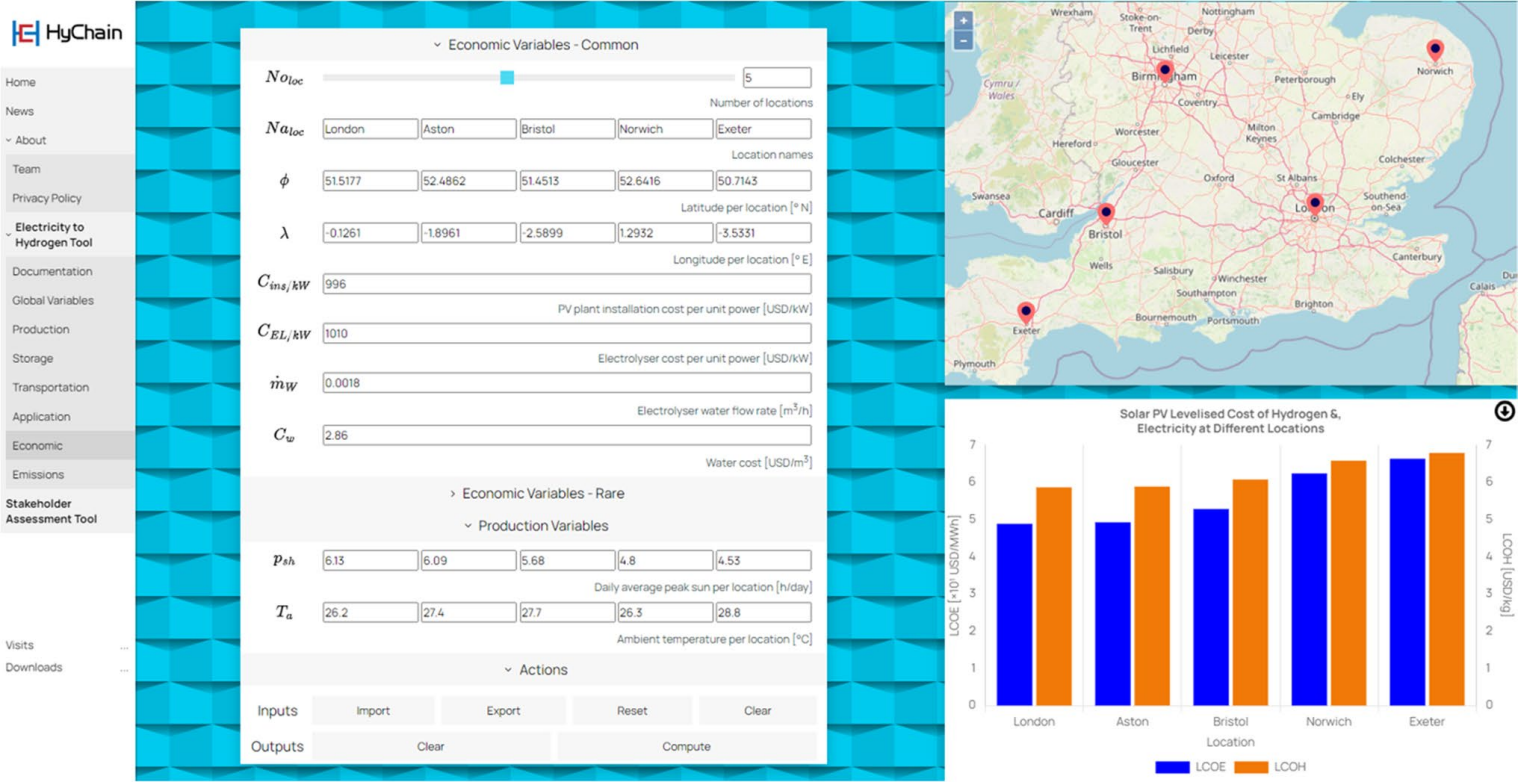
Web application ‘Electricity to Hydrogen Tool’ built in HyChain platform (Economic subpage)

The navigation bar on the left of the website can direct users to the tool. The ‘Electricity to Hydrogen Tool’ is an electricity-to-hydrogen whole chain simulator from green hydrogen production to its storage, transportation, and eventual end-use applications. The backend simulation models have been developed and coded in the Simulink MATLAB software as introduced in the previous sections. The simulation of these four processes in the hydrogen supply chain can be found in the four subpages of ‘Production’, ‘Storage’, ‘Transportation’, and ‘Application’, respectively. Figure 9 shows a screenshot of the ‘Production’ page, where users can fill out the input boxes in the frontend with different values and then click the compute button, it returns on the right-hand side a result diagram. Two performance criteria are tracked, where the LCOE and LCOH results based on different locations are generated in the ‘Economic’ page (see the screenshot in Fig. 10), and the carbon emissions are compared in the ‘Emissions’ page. The web application allows users to import input data and export output data via Microsoft Excel spreadsheet as well.

## Conclusion

The case study on Oman’s green hydrogen supply chain concludes that hydrogen is a feasible and beneficial alternative to traditional fuels such as LPG for cooking in residential sectors. It highlights hydrogen’s advantages in energy efficiency, emission reduction, and sustainability. The study utilised simulation models covering production, storage, transportation, and application phases, factoring in solar power production, storage capacity, pipeline infrastructure, and household energy consumption. It was found that a 7 GW solar plant could produce over 20 times the energy needed for cooking in Muscat households. The study also emphasises hydrogen’s lower greenhouse gas emissions compared to LPG, despite some emissions from production and storage. The levelized costs of the hydrogen supply chain are reasonable and align with other scientific findings. The study suggests that with government support and investment in infrastructure and technology, hydrogen could be used as a cleaner, more sustainable cooking fuel, aiding Oman in achieving its renewable energy and emission reduction goals.

## Supplementary Information

Below is the link to the electronic supplementary material.

Supplementary file1 (DOCX 1531 KB)

## Data Availability

All data generated or analysed during this study are included in this published article and its supplementary information files.

## Nomenclature

${C}_\frac{EL}{kW}$: Electrolyser cost per kW [USD/kW]
${C}_\frac{ins}{kW}$: PV plant installation cost [USD/kW]
${C}_{HS}$: Hydrogen storage cost [USD/kg]
${C}_{OM}$: PV plant operating and maintenance cost [USD/kW]
${C}_{W}$: Water cost [USD/${{\text{m}}}^{3}]$
$${C}_{x}$$: Cost [USD]
${D}_{f}$: Overall derating factor
${D}_{nom}$: Pipe nominal diameter [in]
${D}_{sys}$: System component derating factor
${E}_{EL}$: Electrolyser energy required for one unit volume of hydrogen production [${{\text{kWh}}/{\text{Nm}}}^{3}$]
${E}_{PV}$: Solar PV energy output [kWh]
$${H}_{PV,N{m}^{3}}$$: Hydrogen production [$${Nm}^{3}$$]
${H}_{PV,kg}$: Hydrogen production [kg]
${{\text{LHV}}}_{{H}_{2}}$: Hydrogen energy density (low heating value) [MJ/kg]
${L}_{{\text{Total}}}$: Total pipe distance [km]
${\dot{m}}_{W}$: Electrolyser water flow rate [${{\text{m}}}^{3}$/h]
${{\text{NCV}}}_{{H}_{2}}$: Hydrogen energy net calorific value [MJ/kg]
${{\text{NCV}}}_{NG}$: Natural gas net calorific value [MJ/kg]
${N}_{d}$: Number of days in a period [day]
${P}_{Str}$: Storage pressure [bar]
${P}_{r,PV}$: PV plant rated power output [kW]
${P}_{sh}$: Daily average peak sun hour [h/day]
${S}_{S}$: Solar intensity [kW/${{\text{m}}}^{2}]$
$${T}_{Str}$$: Storage temperature [℃]
$${T}_{a}$$: Ambient temperature [℃]
$${U}_{f}$$: Utilisation factor [%]
$${Vol}_{Str}$$: Storage volume [$${m}^{3}]$$
${d}_{y}$: Days in a year [days]
${d}_{y}$: Days in a year [days]
${n}_{Ele}$: Electrolyser lifetime [years]
${n}_{PV}$: PV panel lifetime [years]
${n}_{Pipe}$: Pipe lifetime [years]
${t}_{st}$: Storage release period per unit of time
${t}_{st}$: Storage release period per unit of time [day]
$${\eta }_{{H}_{2}}$$: Hydrogen efficiency [%]
${\eta }_{NG}$: Natural gas efficiency [%]
${\eta }_{pc}$: PV plant and electrolyser interfacing power converter efficiency [%]
$${\rho }_{H2}$$: Hydrogen density *[*$$kg/{m}^{3}$$]
${\rho }_{Med}$: Hydrogen medium volumetric density [${{{\text{kgH}}}_{2}/{\text{m}}}^{3}]$
Availability: Pipe availability [%]
C_p: Fuel net calorific value [MJ/kg]
Capacity: Pipe capacity [${{\text{kgH}}}_{2}/{\text{day}}]$
*E*: Energy demand [MJ]
EL: Electrolyser
GHG: Greenhouse gas
GWP: Global warming potential
HS: Hydrogen storage [USD]
*i*: Discount rate [%]
ins: Installation
*m*: Total fuel consumption [kg]
mis: Miscellaneous
OM: Operating and maintenance
pc: Power converter
PV: Photovoltaic panel
randi: Volume attribute range
${{\text{CAPEX}}}_{{\text{Pipe}}}$: Pipes capital expenditures [USD/year]
${{\text{CRF}}}_{x}$: Cost recovery factor
${{\text{Cons}}}_{{\text{Cook}}}$: Household cooking energy consumption [MJ/day]
${{\text{Ems}}}_{\frac{{{\text{N}}}_{2}{\text{O}}}{{\text{NG}}}}$: Natural gas ${{\text{N}}}_{2}{\text{O}}$ emissions [g/kg]
${{\text{Ems}}}_{\frac{{{\text{CH}}}_{4}}{{\text{NG}}}}$: Natural gas ${{\text{CH}}}_{4}$ emissions [g/kg]
${{\text{Ems}}}_{\frac{{{\text{CO}}}_{2}}{{\text{NG}}}}$: Natural gas ${{\text{CO}}}_{2}$ emissions [g/kg]
${{\text{Ems}}}_{{{\text{CO}}}_{2}{\text{e}}}$: Carbon dioxide equivalent GHG emission [g/kg]
${{\text{Ems}}}_{{\text{GHG}}/{{\text{H}}}_{2}}$: Hydrogen GHG emissions [g ${{\text{CO}}}_{2}{\text{e}}$ /kg]
$Em{s}_{Total}$: Total GHG emission [g $C{O}_{2}e$/kg]
${{\text{LCOH}}}_{{\text{Comp}}}$: Components levelised cost of hydrogen [${\text{USD}}/{{\text{kgH}}}_{2}$]
${{\text{LCOH}}}_{{\text{Pipe}}}$: Pipe levelised cost of hydrogen [${\text{USD}}/{{\text{kgH}}}_{2}$]
$LCO{H}_{Pipe-System}$: Pipe-system levelised cost of hydrogen [$USD/kg{H}_{2}$]
${\text{LCOE}}$: Levelised cost of electricity [USD/kWh]
${\text{LCOH}}$: Levelised cost of hydrogen [USD/kg]
$M{t}_{{\text{Base}}}$: Material base cost [USD]
$$N{a}_{s}$$: Sample name
$N{o}_{Hshd}$: Number of households
$\mathrm{NonEnergy OPE}{{\text{X}}}_{{\text{Pipe}}}$: Pipe operational expenditures that do not require energy [USD/year]
