# Supplementary material for: Simulation and feasibility assessment of a green hydrogen supply chain: a case study in Oman
Source: Environ Sci Pollut Res Int. 2024 Mar 2;32(22):13313–28. doi: 10.1007/s11356-024-32563-z (PMC12167263; doi:10.1007/s11356-024-32563-z)
Supplement: Supplementary file 1 — Supplementary file1 (DOCX 1531 KB) [file 11356_2024_32563_MOESM1_ESM.docx]

**Design and Feasibility Assessment of a Green Hydrogen Supply Chain: A Case Study in Oman**

Mi Tian*, Shuya Zhong, Muayad Ahmed Mohsin Al Ghassani, Lars Johanning, Voicu Ion Sucala

# The scenario


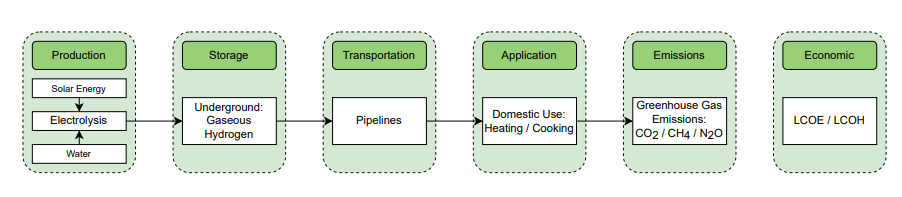


Figure S 1 Simplified Green Hydrogen Supply Chain Flowchart Scenario 2A

# Flowchart of the Algorithm in MatLab


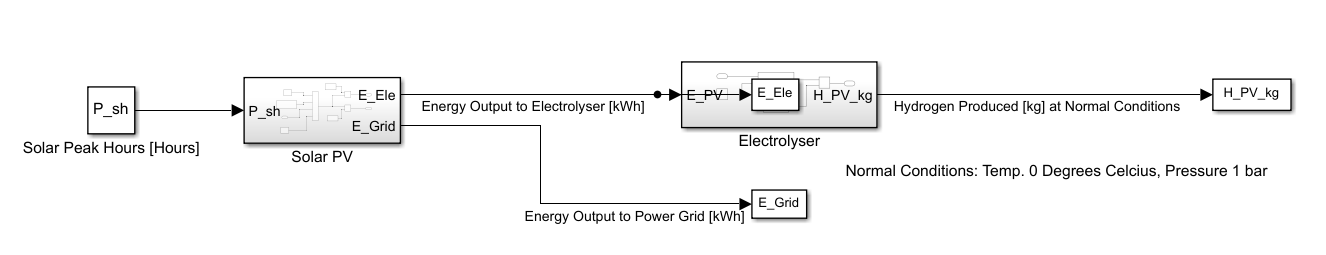


Figure S 2 Production Model


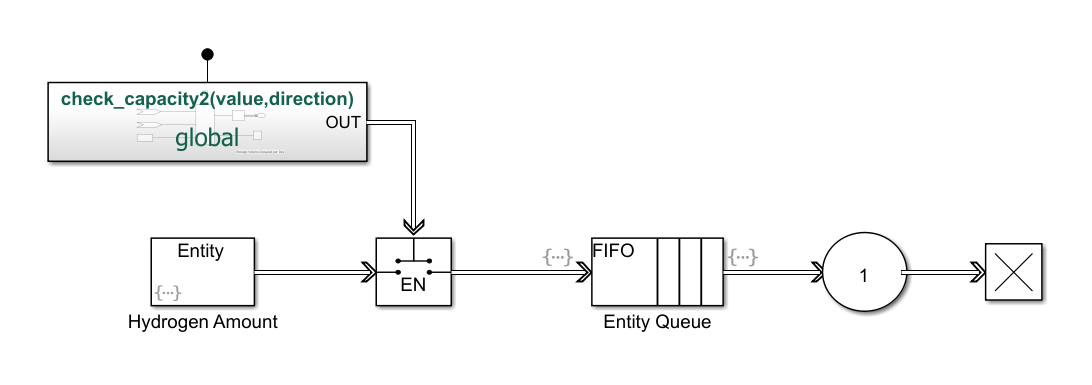


Figure S 3 Storage Model


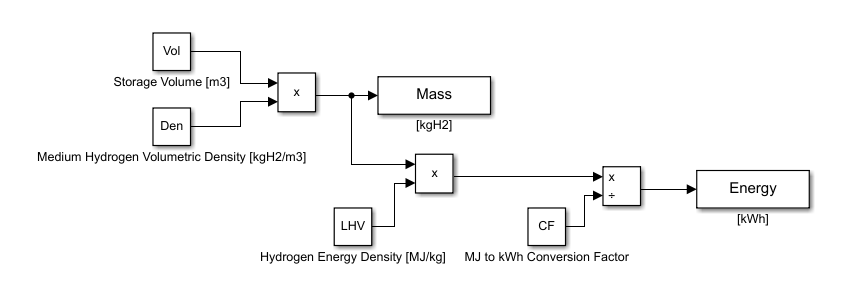


Figure S 4 Storage Medium Model


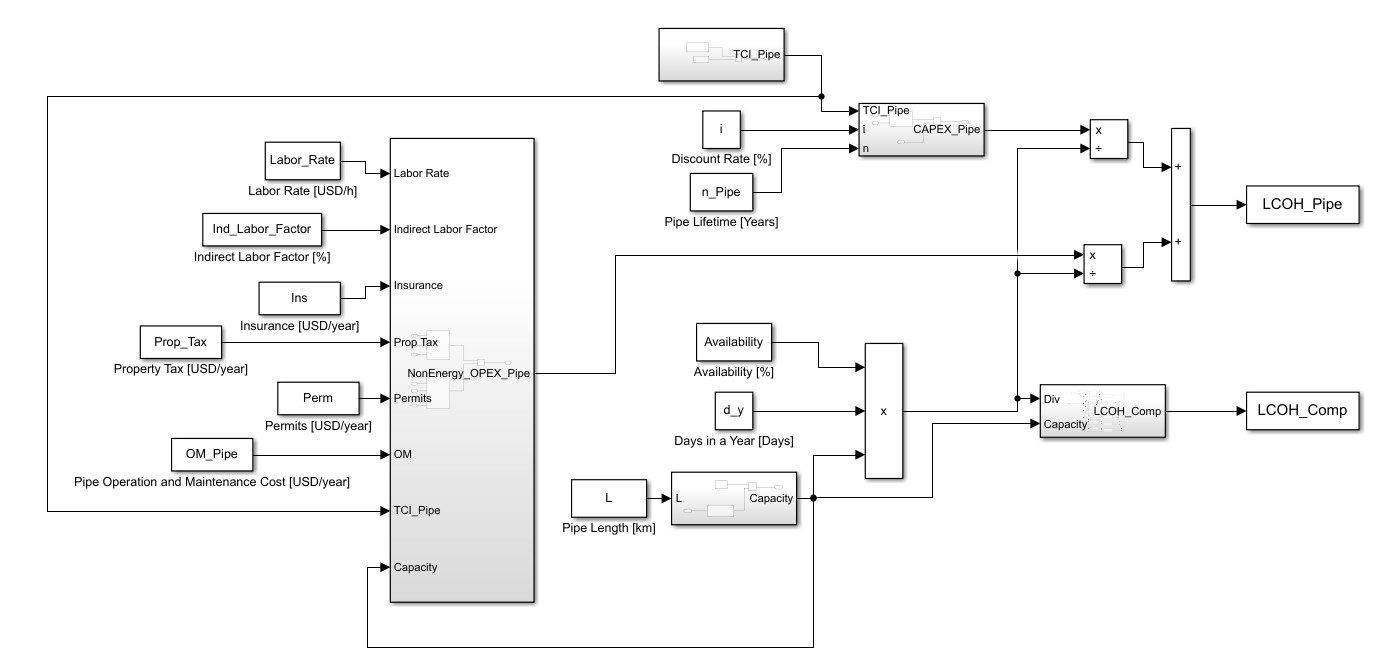


Figure S 5 Transportation Model


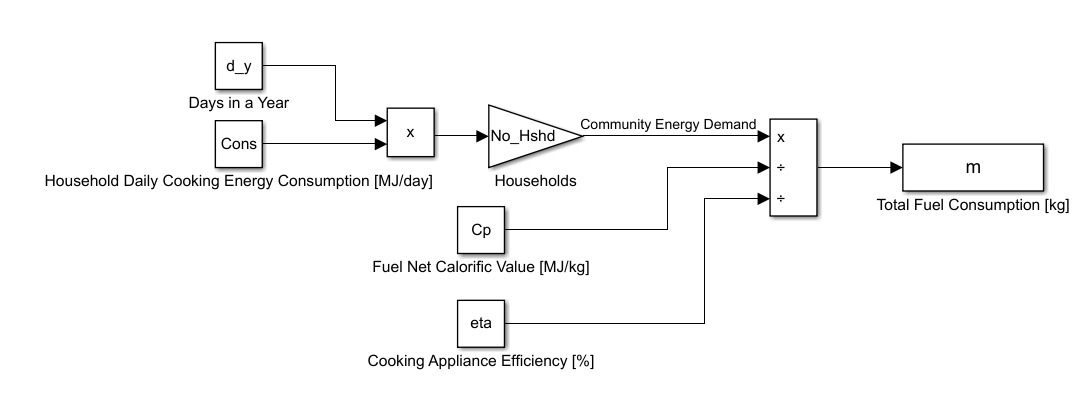


Figure S 6 Application Model


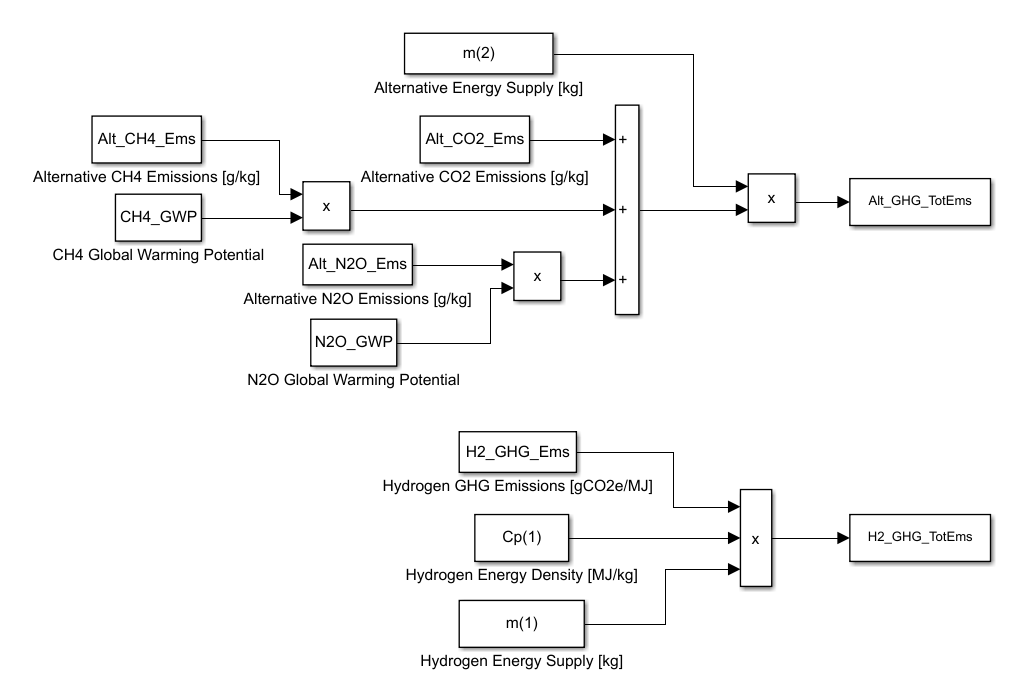


Figure S 7 Greenhouse Gas Emissions Model


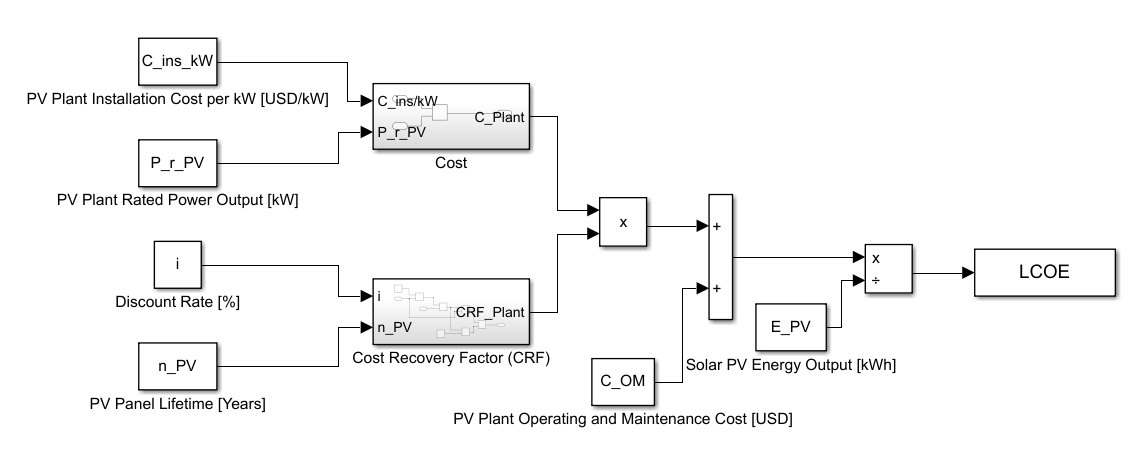


Figure S 8 LCOE Model


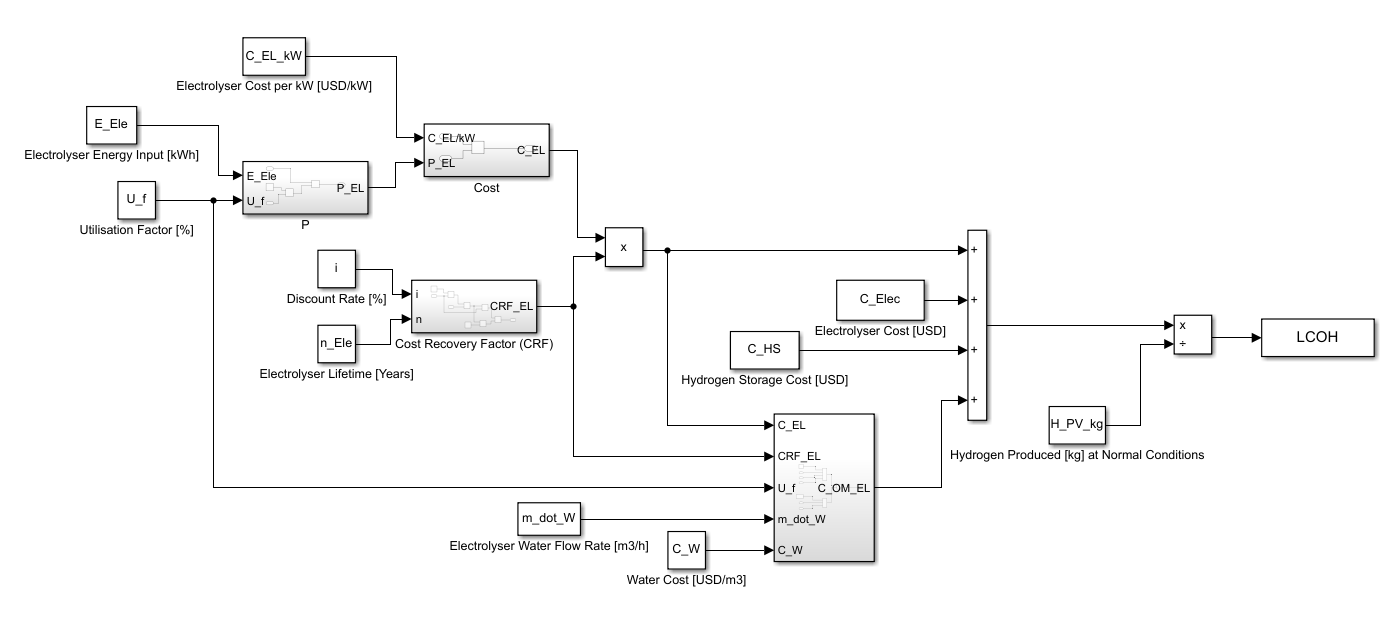


Figure S 9 LCOH Model

# Model Validation

## Production Model Validation

Table S 1 Production Model Validation Parameters

| Parameter | Value | Description | Reference |
| --- | --- | --- | --- |
| $P_{r,PV}$ | 2000 kW | PV Plant Rated Power Output | [1] |
| $P_{sh}$ | [5.68, 5.72, 5.37, etc.] h/day | Daily Average Peak Sun Hour |  |
| $T_{a}$ | [27.7, 27.8, 28.3, etc.] ℃ | Ambient Temperature |  |
| $N_{d}$ | 365 Days | Number of Days in a Period |  |
| $D_{sys}$ | 0.7895 | System Components Derating Factor |  |
| $S_{S}$ | 1 kW/$m^{2}$ | Solar Intensity |  |
| $\eta_{pc}$ | 95 % | PV Plant and Electrolyser Interfacing Power Converter Efficiency |  |
| $E_{EL}$ | 4.53 kWh/$Nm^{3}$ | Electrolyser Energy Required for One Unit Volume of Hydrogen Production |  |
| $\frac{1}{\rho_{H2}}$ | 11.1 $m^{3}/kg$ | Hydrogen Density Reciprocal at Normal Conditions |  |

To validate the production model, Figure 11 illustrates the annual hydrogen production in Oman as computed by the model. The graph aligns closely with the results of the previously mentioned study [1], indicating production values between 50,000 and 70,000 kg for an equivalent 2000 kW solar farm. As a result, the production model is deemed appropriate for application in a novel scenario.


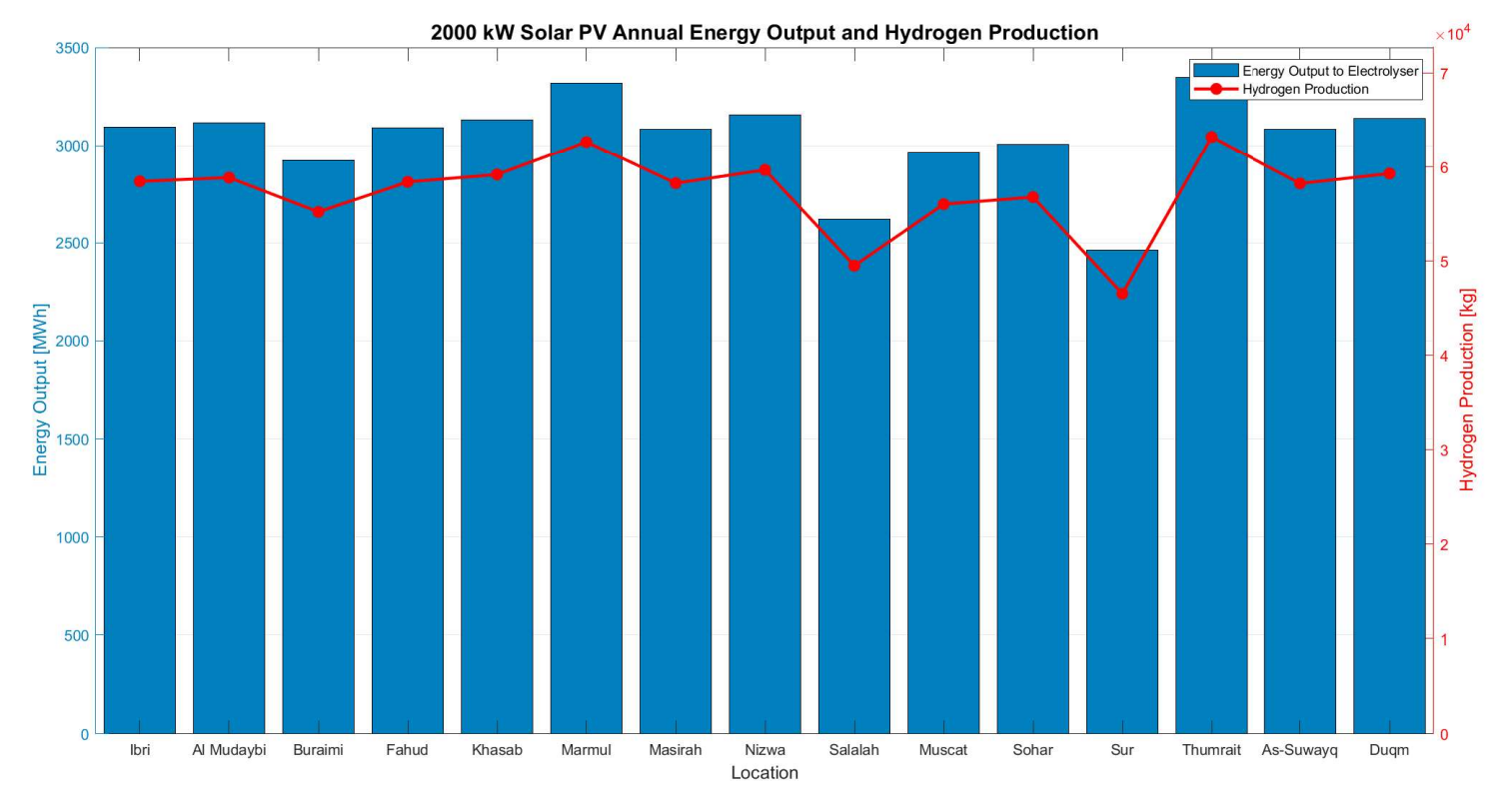


Figure S 10 Hydrogen Production Validation Result

## Storage Model Validation

The storage model, designed to simulate bottle volume within a storage tank, necessitates detailed specifications concerning capacity and the volume of inflowing gas, as outlined in Table S 2.

Table S 2 Storage Model Validation Parameters

| Parameter | Value | Description | Reference |
| --- | --- | --- | --- |
| $randi$ | [1 10] | Volume Attribute Range | [6] |
| ${Vol}_{Str}$ | 50 $m^{3}$ | Storage Capacity |  |
| $t_{st}$ | 8 | Storage Release Period per Unit of Time |  |
| $\rho_{Med}$ | [123, 99, 84, 84, 70, 14.7] ${kgH_{2}/m}^{3}$ | Hydrogen Medium Volumetric Density | [2] |
| ${LHV}_{H_{2}}$ | 120 MJ/kg | Hydrogen Energy Density (Low Heating Value) | [5] |

The model replicates a pattern strikingly akin to the original Figure S 11(left). It simulates gas storage for bottles over a duration of 100 seconds, allocating to each bottle a random volume attribute that spans between 1 and 10. The discharge time for each bottle is set at 8 seconds, while the storage capacity is defined as 50 volume units. The replicated model displayed below manifests a similar pattern when provided with the same inputs. This substantiates the model's adaptability and potential for minor modifications to simulate hydrogen storage. Such modifications would involve integrating values derived from the production model within a variable range, and implementing specific operating conditions to transmute the mass of hydrogen into a volume appropriate for storage.


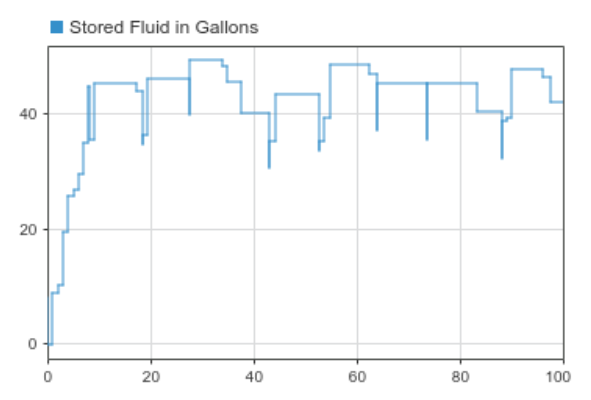

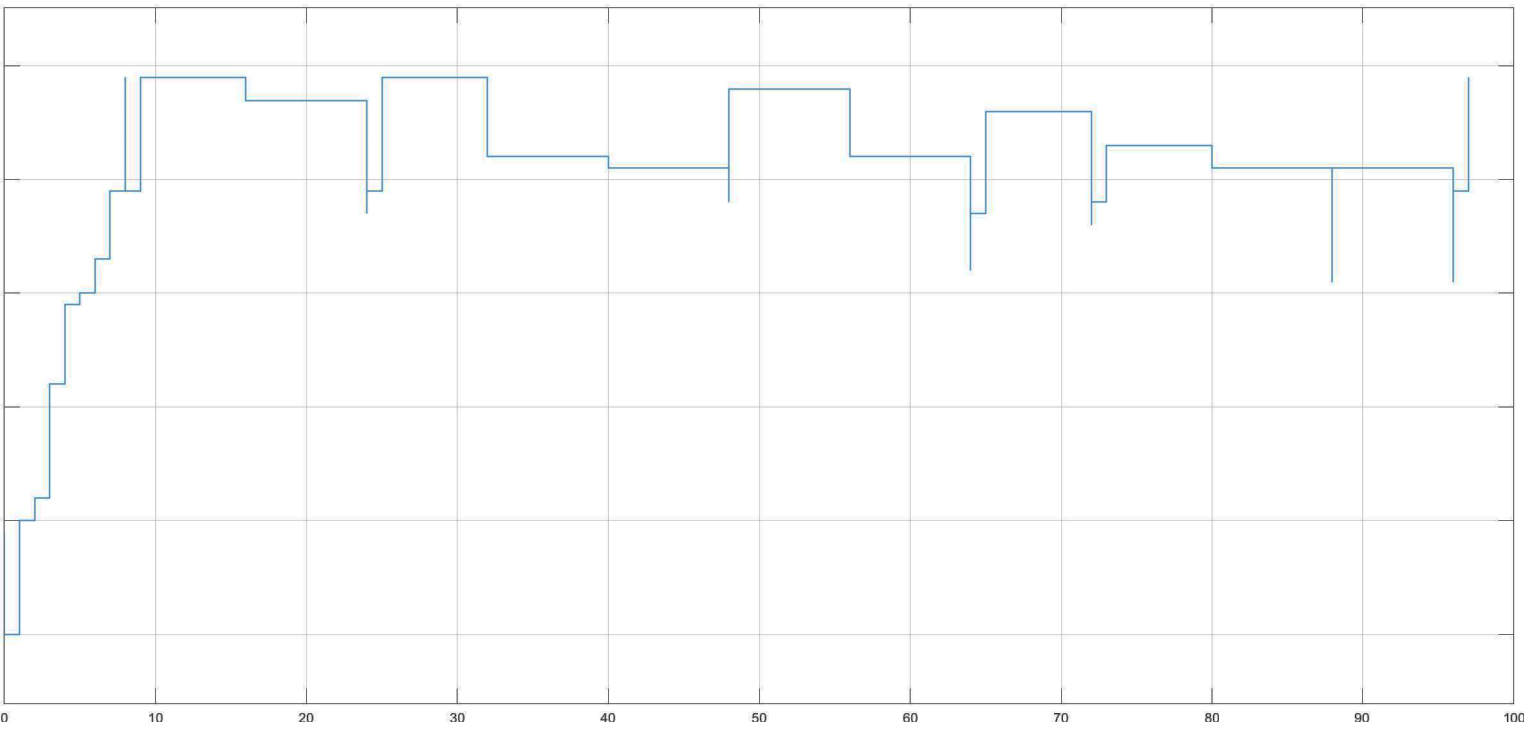


Figure S 11 (left) Storage model referenced result [6] and (right) storage validation result.

The model crafted for various hydrogen storage mediums is corroborated by the analogous results presented in Table S 3 and the referenced study. The selected mediums encompass ammonia, methanol, metal hydrides, liquid hydrogen, and gaseous hydrogen, their respective operating conditions are delineated in Figure S 12. The storage volume and volumetric densities, in conjunction with hydrogen energy density, inform the calculation of the energy yield for each medium. The energy or hydrogen storage capacity per medium aligns closely with the above data, with ammonia exhibiting the highest energy yield, metal hydrides presenting equivalent values, and gaseous hydrogen showing the lowest energy yield. Consequently, given a predefined storage capacity, the energy yield per medium can be simulated in the subsequent section. This will facilitate a discussion pertaining to the energy requirements for seasonal storage.

Table S 3 Storage Model Operating Conditions [2]

| Storage Medium | Pressure [bar] | Temperature [℃] |
| --- | --- | --- |
| Ammonia | 250 | 400 |
| Methanol | 50 | 250 |
| Magnesium Hydride | 30 | 300 |
| Aluminium Hydride | 50 | 1 |
| Liquid Hydrogen | -253 | 1 |
| Hydrogen Gas | 200 | 20 |


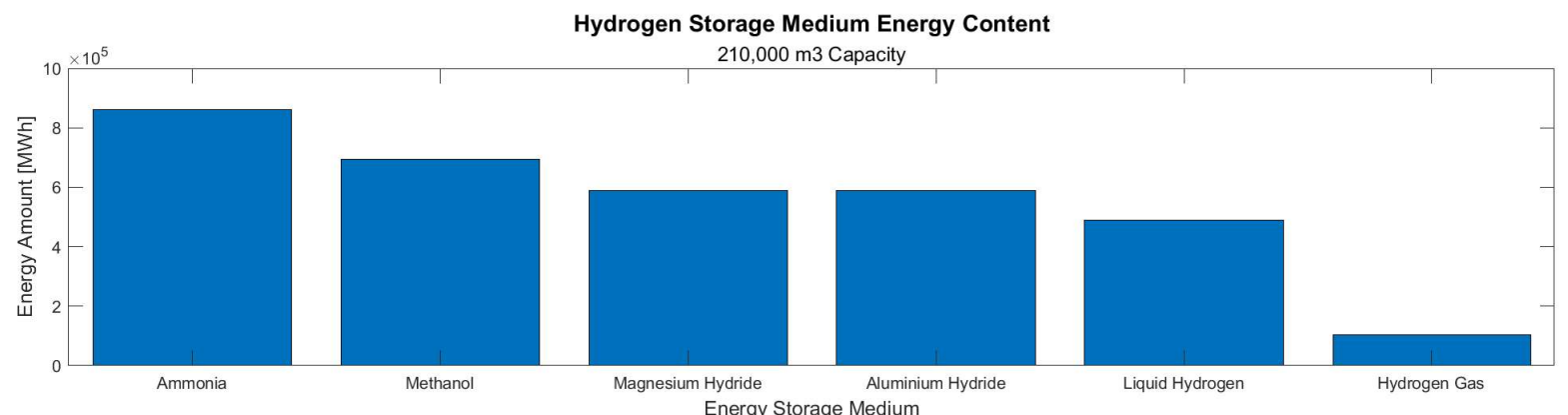


Figure S 12 Hydrogen Storage Medium Validation Result

## Transportation Model Validation

The transportation model's validity is confirmed using the data outlined in Table S 4, and the corresponding results are displayed in Figure S 13. This model emphasizes a singular pipe diameter, specifically 36 inches. The bar graph reveals LCOH values fluctuating between 0.8 and 0.9 USD/kg, aligning with expectations based on the technical brief. As indicated, pipeline costs diminish, while compressor costs increase with reduced distances between them. Consequently, the model is deemed valid and is slated for application in a distinct scenario in the subsequent section.

Table S 4 Transportation Pipe Model Validation Parameters

| Parameter | Value | Description | Reference |
| --- | --- | --- | --- |
| i | 8 % | Discount Rate | [4] |
| $n_{Pipe}$ | 50 Years | Pipe Lifetime |  |
| $L_{Total}$ | 1500 km | Total Pipe Distance |  |
| $Mt_{Base}$ | 63,027 USD | Material Base Cost |  |
| $D_{nom}$ | 36 in | Pipe Nominal Diameter |  |


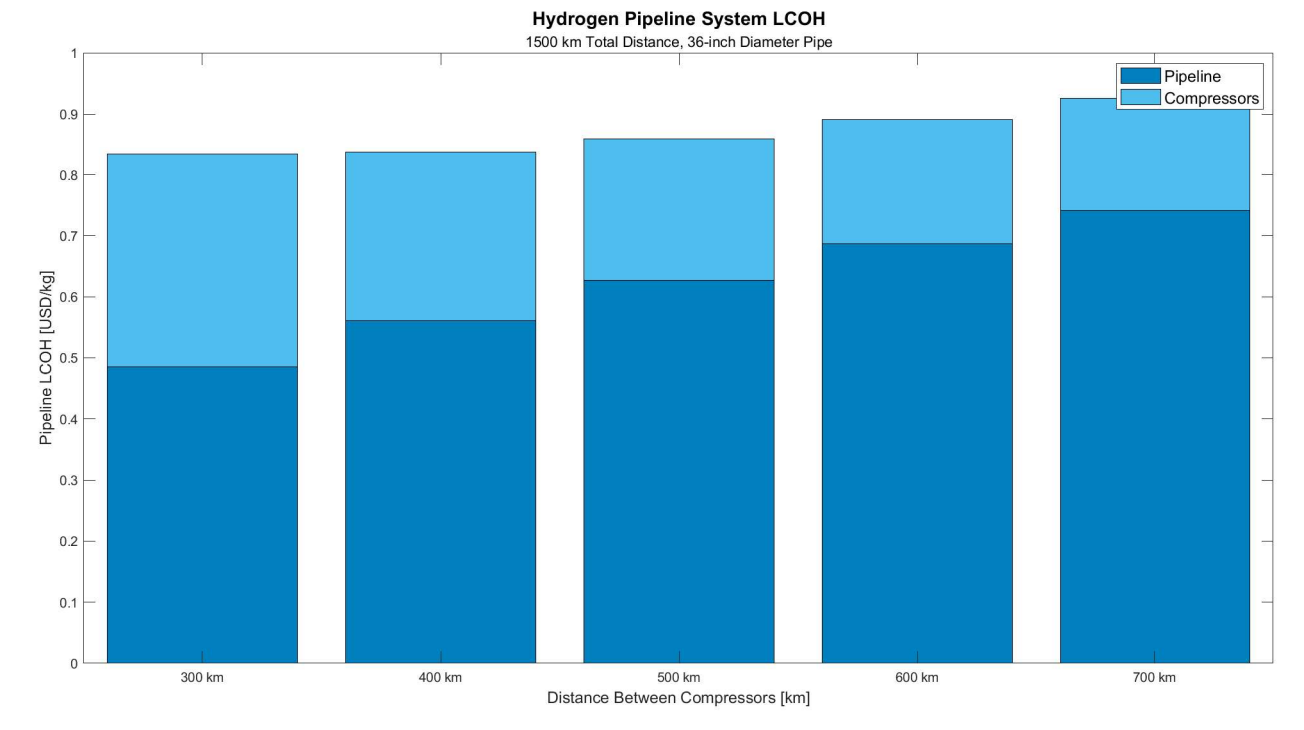


Figure S 13 Hydrogen Pipeline Transportation Validation Result

## Application Model Validation

The employed model utilizes data from Table S 5to simulate the energy quantity of each fuel necessary to meet demand. The resultant Figure S 14 indicates that less hydrogen is needed to satisfy the demand in the given scenario compared to natural gas, which aligns with the study's findings. Furthermore, by building on the production model, the quantity of hydrogen produced can be compared with the hydrogen demand for a scenario. This facilitates an effective comparison to ascertain the number of households that can be satisfied, or the capacity of the solar farm to support a specific number of households.

Table S 5 Application Model Validation Parameters

| Parameter | Value | Description | Reference |
| --- | --- | --- | --- |
| $No_{Hshd}$ | 20 | Number of Households | [5] |
| $Cons_{Cook}$ | 9 MJ/day | Household Cooking Energy Consumption |  |
| $d_{y}$ | 365 Days | Days in a Year |  |
| $\eta_{NG}$ | 38 % | Natural Gas Efficiency | [7] |
| $\eta_{H_{2}}$ | 60 % | Hydrogen Efficiency | [5] |
| ${NCV}_{H_{2}}$ | 120 MJ/kg | Hydrogen Energy Net Calorific Value |  |
| ${NCV}_{NG}$ | 55 MJ/kg | Natural Gas Net Calorific Value | [8] |


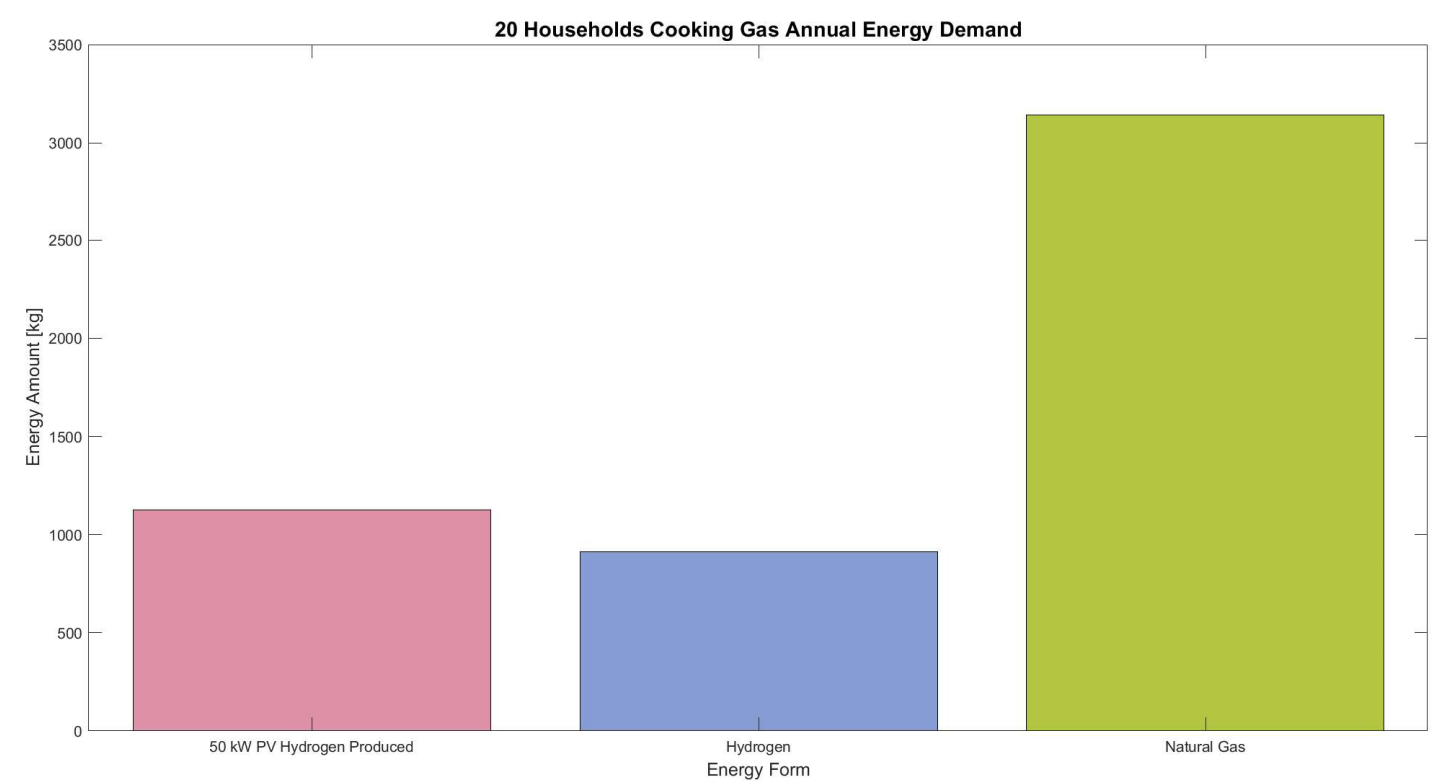


Figure S 14 Hydrogen Cooking Application Validation Result

## Emissions Model Validation

The emissions model's validity was confirmed using data from the 2022 US EPA's Greenhouse Gas Inventories, shown in Table S 6, which detail the environmental impact of emissions resulting from fuel consumption. This data was incorporated into a model to compare greenhouse gas emissions from fuels, including CO_2_, CH_4_, and NO_2_. Figure S 15 substantiates the expectation that hydrogen emits fewer emissions than natural gas—nearly four to five times less, representing a substantial reduction. However, given that green hydrogen is derived from renewable sources, the majority of emissions stem from the compression and transportation phases.

Table S 6 Emissions Model Validation Parameters

| Parameter | Value | Description | Reference |
| --- | --- | --- | --- |
| $Ems_{CO_{2}/NG}$ | 2766 g/kg | Natural Gas $CO_{2}$ Emissions | [9] |
| $Ems_{CH_{4}/NG}$ | 52.13 g/kg | Natural Gas $CH_{4}$ Emissions |  |
| $Ems_{N_{2}O/NG}$ | 5.213 g/kg | Natural Gas $N_{2}O$ Emissions |  |
| $Ems_{GHG/H_{2}}$ | 40.2 g$CO_{2}e$ /kg | Hydrogen GHG Emissions | [5] |


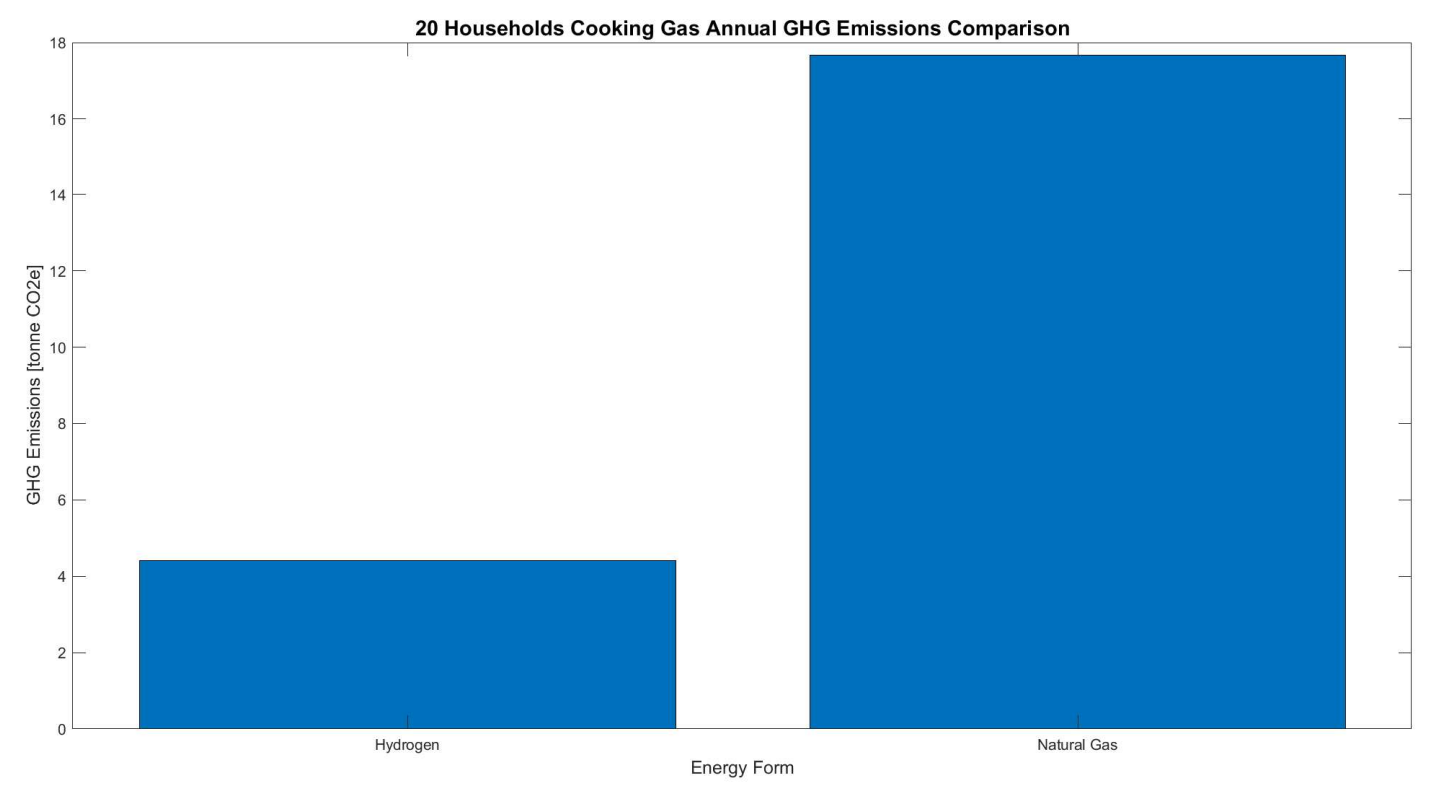


Figure S 15 Hydrogen Cooking Emissions Validation Result

## Levelised Cost Model Validation

The Levelized Cost of Hydrogen (LCOH) was simulated in the study by Ahshan (2021), with values ranging from 6.31 to 7.32 USD/kg listed in Table S 7. The validity of the LCOH is confirmed by its similarity to the results depicted in Figure S 16. However, the modelled values present a smaller range, with a variance of 5 - 10%. This outcome establishes the model's appropriateness for application in a different scenario in the subsequent section.

Table S 7 Levelised Cost Model Validation Parameters

| Parameter | Value | Description | Reference |
| --- | --- | --- | --- |
| $C_{ins/kW}$ | 996 USD/kW | PV Plant Installation Cost | [1] |
| $i$ | 6.5 % | Discount Rate |  |
| $n_{PV}$ | 25 Years | PV Panel Lifetime |  |
| $C_{OM}$ | 10 USD/kW | PV Plant Operating and Maintenance Cost |  |
| $C_{EL/kW}$ | 1010 USD/kW | Electrolyser Cost per kW |  |
| $U_{f}$ | 20 % | Utilisation Factor |  |
| $n_{Ele}$ | 20 Years | Electrolyser Lifetime |  |
| $C_{HS}$ | 0.5 USD/kg | Hydrogen Storage Cost |  |
| $OM_{Perc}$ | 3 % | Operation and Maintenance Cost |  |
| $ṁ_{W}$ | 0.0018 $m^{3}$/h | Electrolyser Water Flow Rate | [10] |
| $C_{W}$ | 2.86 USD/$m^{3}$ | Water Cost | [11] |


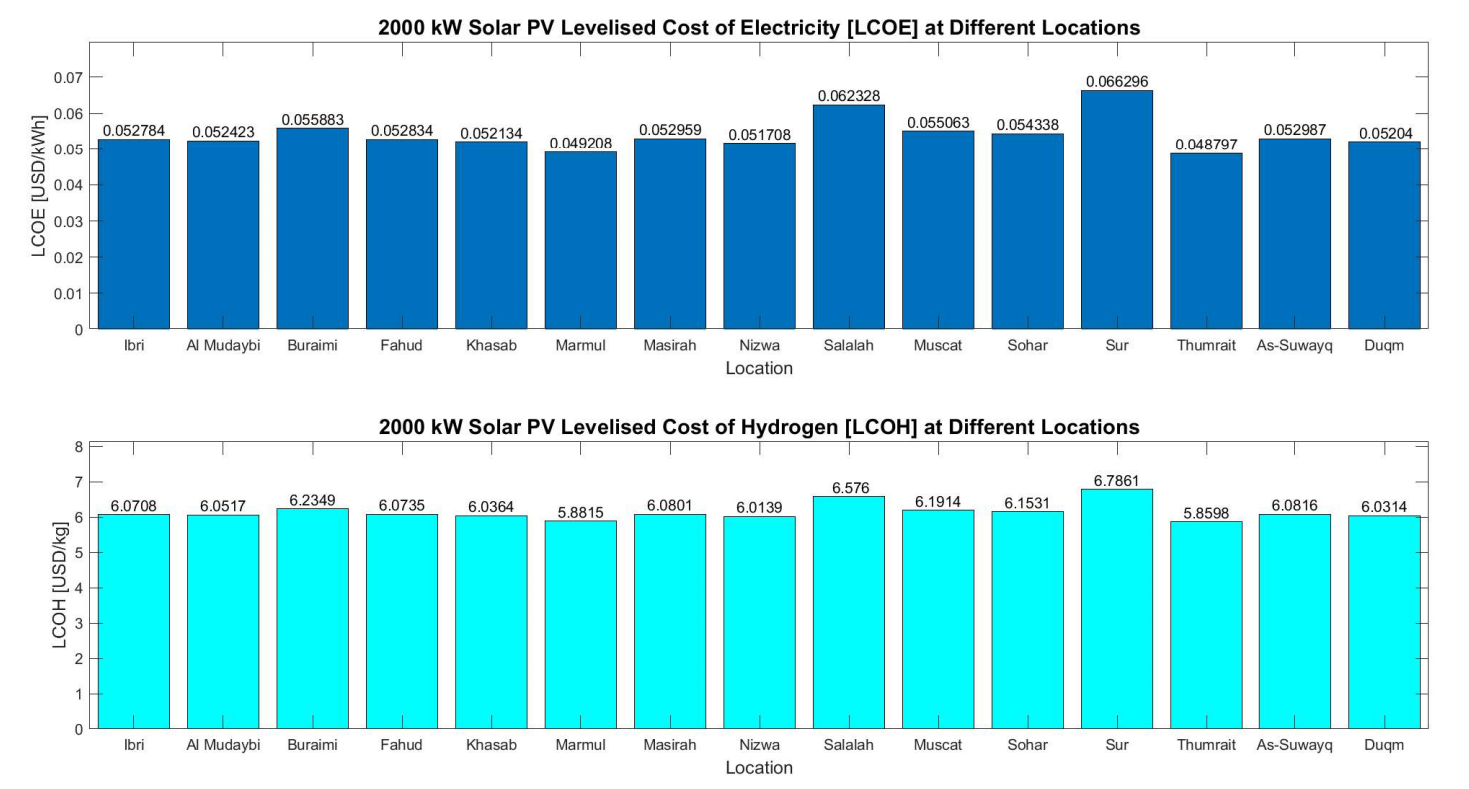


Figure S 16 Levelised Cost of Hydrogen Validation Result
